# Homoleptic Rare-Earth-Metal Sandwiches with Dibenzo[*a*,*e*]cyclooctatetraene Dianions

**DOI:** 10.1021/acs.inorgchem.3c04249

**Published:** 2024-02-20

**Authors:** Yikun Zhu, James Mahoney, Aaron J. Babson, Zheng Zhou, Zheng Wei, Miguel Gakiya-Teruya, James McNeely, Andrey Yu. Rogachev, Michael Shatruk, Marina A. Petrukhina

**Affiliations:** † Department of Chemistry, University at Albany, State University of New York, Albany, New York 12222, United States; ‡ Interdisciplinary Materials Research Center, School of Materials Science and Engineering, Tongji University, Shanghai 201804, China; § Department of Chemistry and Biochemistry, Florida State University, Tallahassee, Florida 32306, United States; ∥ Department of Chemistry, Boston University, Boston, Massachusetts 02215, United States; ⊥ Department of Chemistry, Illinois Institute of Technology, Chicago, Illinois 60616, United States

## Abstract

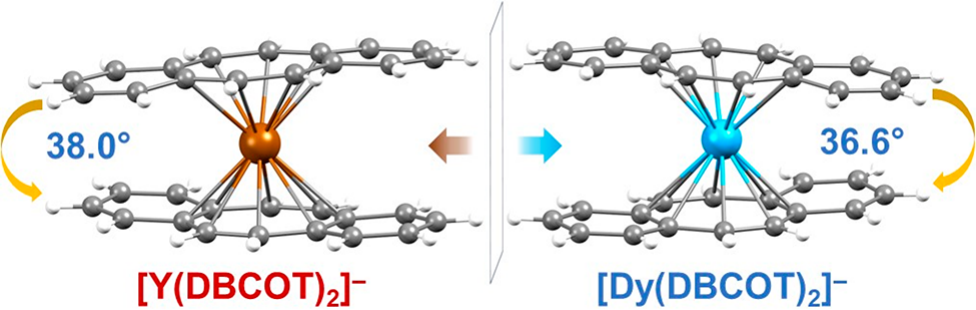

A family of rare-earth complexes [RE(III) = Y, La, Gd, Tb, Dy, and Er] with doubly reduced dibenzo[*a*,*e*]cyclooctatetraene (DBCOT) has been synthesized and structurally characterized. X-ray diffraction analysis confirms that all products of the [RE(DBCOT)(THF)_4_][RE(DBCOT)_2_] composition consist of the anionic sandwich [RE(DBCOT)_2_]^−^ and the cationic counterpart [RE(DBCOT)(THF)_4_]^+^. Within the sandwich, two elongated π decks are slightly bent toward the metal center (avg. 7.3°) with a rotation angle of 35.9–37.6°. The RE(III) ion is entrapped between the central eight-membered rings of DBCOT^2–^ in a η^8^ fashion. The trends in the RE–COT bond lengths are consistent with the variations of the ionic radii of RE(III) centers. The ^1^H NMR spectra of the diamagnetic Y(III) and La(III) analogues illustrate the distinct solution behavior for the cationic and anionic parts in this series. Magnetic measurements for the Dy analogue reveal single-molecule magnetism, which was rationalized by considering the effect of crystal-field splitting for both building units analyzed by electronic structure calculations.

## Abstract 2

A new class of DBCOT-based homoleptic rare-earth-metal sandwiches is synthesized and structurally characterized for the series RE = Y, La, Gd, Tb, Dy, and Er, including a Dy-based single-molecule magnet.

## Introduction

**Scheme 1:**
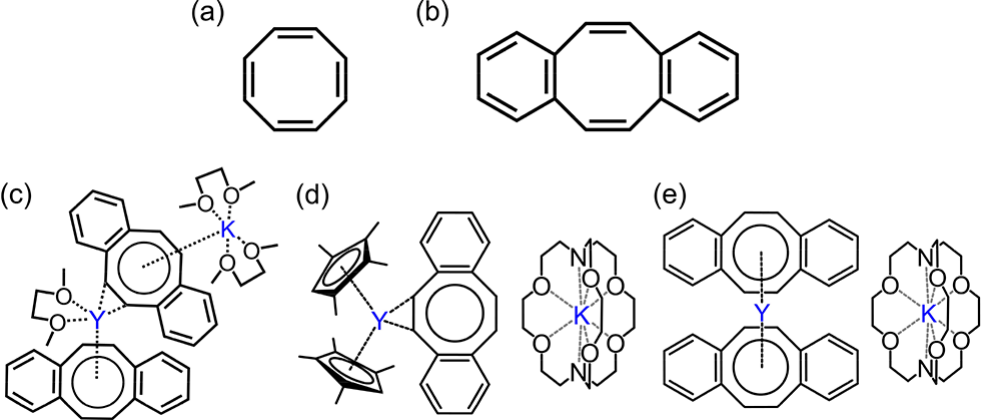
Depictions of COT and DBCOT, along with the Mixed-Metal and Mixed-Ligand Rare-Earth-Metal Complexes of DBCOT^2–^

Cyclooctatetraene (COT; Scheme 1a), a nonplanar tub-shaped molecule with 8π electrons, has attracted significant attention of both the organic and organometallic communities. Since the first report on the chemical reduction of COT by Katz^1^ and structural characterization of the U(COT)_2_ sandwich by Streitwieser,^2^ the COT^2–^ ligand has been widely utilized in the preparation of numerous organometallic complexes with d- and f-block metals. By control of the ligand-to-metal ratio, using mixed-ligand combinations, or the introduction of multiple metal centers, the geometry and composition of the resulting complexes have been broadly modified, significantly affecting their performance in materials applications.^3−9^ Remarkably, COT-based lanthanide complexes with a certain molecular symmetry were shown to exhibit outstanding single-molecule-magnet (SMM) behavior.^10−18^ Moreover, a great number of derivatives with different substituents have been employed in organometallic synthesis,^19,20^ opening additional pathways for tuning magnetic properties through structural modulation.^21,22^ However, functionalization of the COT core has been mainly limited to substituents such as trimethylsilyl or cycloalkyl groups, while larger polyaromatic ligands built around an eight-membered ring have remained largely unexplored.

Specifically, *sym*-dibenzo[*a*,*e*]cyclooctatetraene (DBCOT; Scheme 1b), a π-expanded derivative of COT, was shown to function as an enhanced π-donor for transition-metal catalysis,^23−26^ as well as a redox-active subunit for organic electrode materials.^27^ In our recent work, we reported the chemical reduction behavior of DBCOT with all Group 1 metals and showed that it can readily accept two electrons to form a planarized π-expanded dianion.^28^ Notably, the fusion of two annulated benzene rings to the central COT core provides additional binding sites that result in versatile metal coordination of the doubly reduced DBCOT^2–^ and diverse solid-state packing of the resulting products.

To date, only three types of rare-earth-metal complexes with DBCOT anions have been reported. In 2020, Bloch built the first structure of an yttrium complex, [K(DME)_2_Y(DME)(DBCOT)_2_] (Scheme 1c), with a Y:DBCOT ratio of 1:2, where the Y(III) ion is asymmetrically bound to DBCOT^2–^ in the η^2^ and η^8^ modes.^29^ Very recently, the mixed-ligand (heteroleptic) complexes [K([2.2.2]cryptand)][Cp^tet^_2_RE(DBCOT)] (RE = Y and Dy; Cp^tet^ = tetramethylcyclopentadienyl) were reported by Demir et al.^30^ Those have a Y:DBCOT ratio of 1:1, with the Y(III) ion being η^2^-coordinated to the eight-membered ring of the dianion (Scheme 1d). Importantly, the Dy(III) analogue was shown to be a SMM,^30^ stimulating further research of these systems. Following this work on the mixed-ligand compounds, Demir et al. reported another heterometallic complex, [K([2.2.2]cryptand)][Y(DBCOT)_2_], which is built by a clathrated K^+^ cation and a sandwich-type anion with a Y:DBCOT ratio of 1:2 (Scheme 1e).^31^

Inspired by these recent works, we hypothesized that a homoleptic Dy(III) complex prepared with the doubly reduced DBCOT^2–^ anion could also behave as a SMM. Moreover, it would be of interest to evaluate how the replacement of two (Cp^tet^)^−^ ligands with a single DBCOT^2–^ ligand affects the SMM behavior. In this work, we report the synthesis and structural characterization of new homoleptic sandwich-type complexes of rare-earth metals with the doubly reduced DBCOT. Specifically, a series of complexes with the general formula [RE(DBCOT)(THF)_4_][RE(DBCOT)_2_] (RE = Y, La, Gd, Tb, Dy, and Er) were prepared and fully characterized using single-crystal X-ray diffraction and spectroscopic techniques. Magnetic studies revealed that the Dy complex shows SMM behavior, which was justified by electronic structure calculations.

## Results and Discussion

### Crystallographic Study

**Scheme 2:**
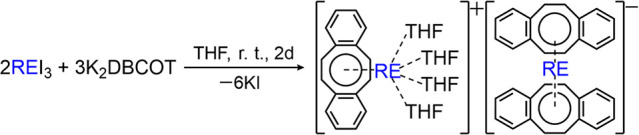
Preparation of Complexes **1**–**6** (RE = Y, La, Gd, Tb, Dy, and Er, Respectively)

The new family was prepared by the reaction of REI_3_ [RE(III) = Y, La, Gd, Tb, Dy, and Er] and K_2_DBCOT salt, similar to our previous synthesis of Ln-based COT complexes.^9^ Specifically, a solution of K_2_DBCOT (1.5 equiv) was added dropwise to a slurry of REI_3_ in tetrahydrofuran (THF) at room temperature, and the reaction was allowed to proceed to completion for 48 h (Scheme 2). After removal of KI salt by filtration, the products were crystallized using slow evaporation of the solvent at elevated temperatures. Brownish block-shaped crystals were isolated in good yield after several days (see the Experimental Section for more details). The X-ray diffraction study confirmed that products of the [RE(DBCOT)(THF)_4_][RE(DBCOT)_2_] composition (**1-Y**, **2-La**, **3-Gd**, **4-Tb**, **5-Dy**, and **6-Er**) are isostructural and conform to a monoclinic *C*2 space group with *Z* = 4 (Table S5). The unit cell volume decreases in the order of **2-La** [5267.7(9) Å^3^] > **3-Gd** [5120.5(6) Å^3^] > **4-Tb** [5093.0(8) Å^3^] > **1-Y** [5068.3(4) Å^3^] > **5-Dy** [5051.6(5) Å^3^] > **6-Er** [5037.0(4) Å^3^], in agreement with the lanthanide contraction. The phase purity of **1** and **2** was proven by ^1^H NMR spectroscopy (Figures S4 and S5) and that of **3**–**6** was confirmed by powder X-ray diffraction (Figures S6–S9 and Tables S1–S4).

**Figure 1:**
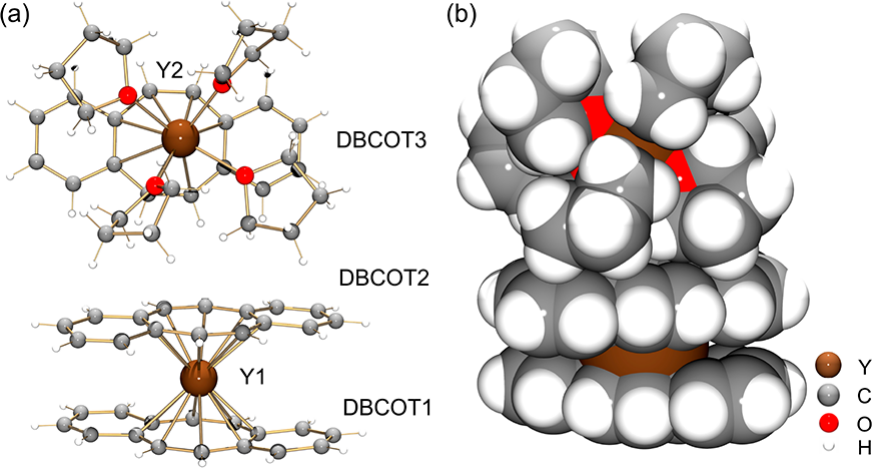
Crystal structure of **1-Y** as (a) ball-and-stick and (b) space-filling models.

**Figure 2:**
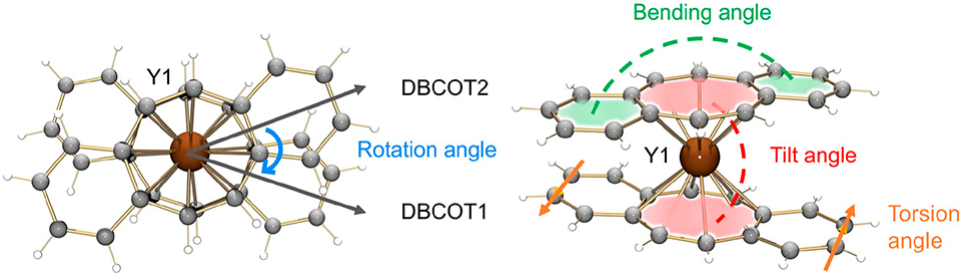
Calculation of selected angles using top and side views of the [Y(DBCOT)_2_]^−^ sandwich.

Due to high similarity of compounds **1**–**6** and very close geometric parameters with the Dy analogue, **1-Y** is selected as the representative complex for the detailed structural description. In the crystal structure of **1-Y** (Figure 1), the Y1 ion is entrapped by two π decks to form an anionic [Y(DBCOT)_2_]^−^ sandwich, which is separated from the cationic half-sandwich [Y(DBCOT)(THF)_4_]^+^. In the sandwich, the Y1 ion is bound to the central eight-membered rings of two DBCOT^2–^ anions in a η^8^ mode, with the Y–C distances spanning a broader range [2.578(3)–2.734(3) Å] in comparison to its COT analogue [2.609(1)–2.669(1) Å],^12^ thus indicating less symmetric coordination toward the eight-membered rings. The Y1 ion is placed slightly closer to DBCOT1 compared to DBCOT2, with Y–DBCOT_centroid_ distances of 1.877(3) Å and 1.889(3) Å, respectively. The two central COT rings are almost parallel to each other with a deck-to-deck separation of 3.766(3) Å. A small tilt of 2.0° between two COT rings (Figure 2) is commonly seen in the [RE(COT)_2_]^−^ moieties.^12,14^ The DBCOT^2–^ dianions are both slightly bent toward the Y(III) center with bending angles of 7.6° and 6.3°, respectively (Figure 2). In contrast to the COT^2–^ units in the [RE(COT)_2_]^−^ moieties,^12,14^ the DBCOT decks are twisted with respect to each other, with a rotation angle of 37.6° (the blue angle in Figure 2), which is larger than that in the columnar structure of [{Na_2_(THF)_3_}(DBCOT)]_∞_ reported previously (7.5°).^28^ As a result, a decrease of the deck-to-deck separation between the two almost parallel central COT rings is observed in **1-Y** [3.766(3) Å] versus that in the [Y(COT)_2_]^−^ analogue [3.811(1) Å].^12^

In the cationic half-sandwich [Y(DBCOT)(THF)_4_]^+^, the Y2 ion is η^8^-coordinated to the central eight-membered ring of one DBCOT^2–^ anion [Y–C: 2.564(3)–2.782(3) Å] and is also capped with four THF molecules [Y–O_THF_: 2.377(2)–2.421(2) Å], thus forming a “piano-stool”-like moiety that is commonly seen in the halogen-bridged dimeric COT complexes.^33^ It is worth noting that the Y2–DBCOT_centroid_ distance is longer [1.924(3) Å] compared to that in the anionic sandwich. Plus, the third DBCOT dianion is bending away from the Y2 counterion and slightly twists along the core length, with bending and torsion angles (the orange angle in Figure 2) of 8.3° and 6.0°, respectively. All Y–C and Y–O distances are comparable to those reported in the literature.^9,11,12^

**Figure 3:**
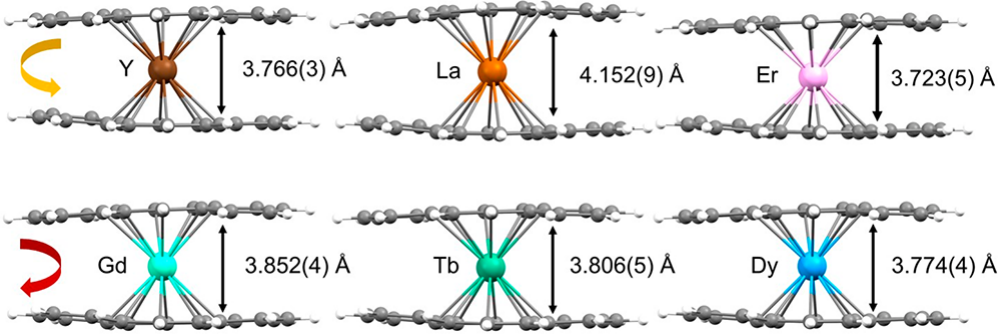
Deck-to-deck separations and rotation direction (counterclockwise vs clockwise) in **1**–**6** in ball-and-stick models.

A detailed structural comparison of the bond distances and angles in complexes **1**–**6** reveals interesting trends. The following order is considered in Table 1 based on decreasing ionic radii: **2-La** > **3-Gd** > **4-Tb** > **5-Dy** > **1-Y** > **6-Er**.^32^ Considering the [RE(DBCOT)_2_]^−^ sandwiches from **2-La** to **6-Er**, the RE1-to-ring-centroid distance is gradually shortened from 2.068(9) to 1.855(5) Å and from 2.084(8) to 1.868(5) Å (for DBCOT1 and DBCOT2, respectively), which is consistent with the decrease of the RE(III) ionic radius. The RE(III) ion sits closer to DBCOT1 than to DBCOT2, with the RE–DBCOT_centroid_ distance difference averaging at 0.013 Å. Consequently, the separation between two DBCOT decks in the homoleptic sandwich units is also notably different and depends on the ionic radius of the metal centers; it is decreased from 4.152(9) Å in **2-La** to 3.723(5) Å in **6-Er** (Figure 3). In the [RE(DBCOT)(THF)_4_]^+^ cation, the RE2-to-ring-centroid distance is also decreased from 2.060(9) to 1.915(4) Å. Notably, the La1–DBCOT_centroid_ distance is longer than the La2–DBCOT_centroid_ distance, while this trend is reversed in other complexes. This indicates that the La(III) ion exhibits weaker binding in such sandwich-based systems, which could explain some differences detected in solution by ^1^H NMR spectroscopy (*vide infra*).

**Table 1:** Selected Distances (Å) and Angles (°) in Complexes **1**–**6**^a^

	**2-La**	**3-Gd**	**4-Tb**	**5-Dy**	**1-Y**	**6-Er**
ionic radii^32^	1.160	1.053	1.040	1.027	1.019	1.004
RE1–DBCOT1_centroid_	2.068(9)	1.920(4)	1.897(5)	1.881(7)	1.877(3)	1.855(5)
RE1–DBCOT2_centroid_	2.084(8)	1.932(4)	1.909(5)	1.893(7)	1.889(3)	1.868(5)
deck-to-deck separation	4.152(9)	3.852(4)	3.806(5)	3.774(7)	3.766(3)	3.723(5)
RE2–DBCOT3_centroid_	2.060(9)	1.945(4)	1.932(5)	1.928(7)	1.924(3)	1.915(4)
RE2–O_THF_	2.533(6)	2.442(12)	2.421(4)	2.415(5)	2.399(2)	2.388(3)
tilt angle_12_	3.1	2.4	2.2	2.2	2.0	2.0
rotation angle_12_	35.9	36.2	36.2	36.3	37.6	37.6
bending angle_1_	9.3	8.0	7.7	7.7	7.6	7.3
bending angle_2_	8.5	6.8	6.3	6.4	6.3	5.8
bending angle_3_	3.9	7.0	7.6	8.6	8.3	10.1
torsion angle_1_	1.3	1.2	1.4	2.0	1.9	2.0
torsion angle_2_	1.4	3.1	3.2	3.8	4.1	3.8
torsion angle_3_	3.0	5.2	5.1	6.3	6.0	8.2

a The subscripts 1–3 for the angles indicate measurements done for the particular DBCOT units, as labeled in Figure 1.

From **2-La** to **1-Y**, the two DBCOT decks in the sandwich can be considered parallel with small deviations (2.0–3.1°), while the rotation angle is slightly increased along the series (35.9–37.6°). Upon complexation, the DBCOT1 and DBCOT2 dianions bend toward the RE1 centers with a slight twist along the core length (1.2–2.0° and 1.4–4.1°, respectively). In contrast, the DBCOT3 dianion is bending away from the RE2 ion and shows the largest twist 8.2° in **6-Er** for the cationic moieties. In addition, the decrease in the rare-earth-metal size is accompanied by planarization of the anionic species (DBCOT1 and DBCOT2), in contrast to the planarity decrease of the cationic part (DBCOT3), as shown in the bending angle trends (Table 1).

**Figure 4:**
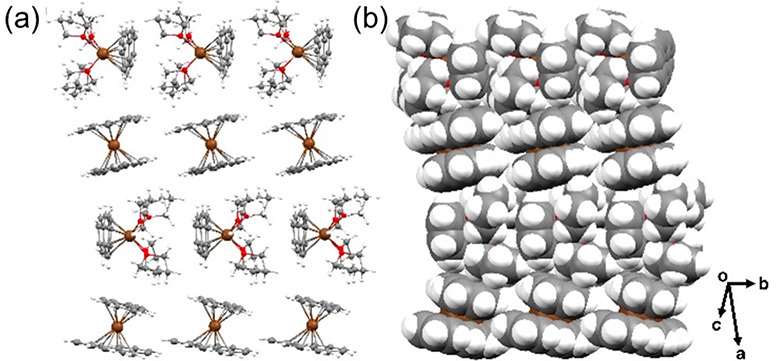
Solid-state packing of **1**–**6** in (a) ball-and-stick and (b) space-filling models.

In the solid-state structures of **1**–**6** (Figure 4), the 2D layers are formed through weak C–H···π interactions [2.591(4)–2.819(5) Å] between the DBCOT^2–^ anions and coordinated THF molecules of the cationic moieties. The shortest intermolecular RE–RE distances between the columns range over 7.577(4)–7.958(9) Å.

**Figure 5:**
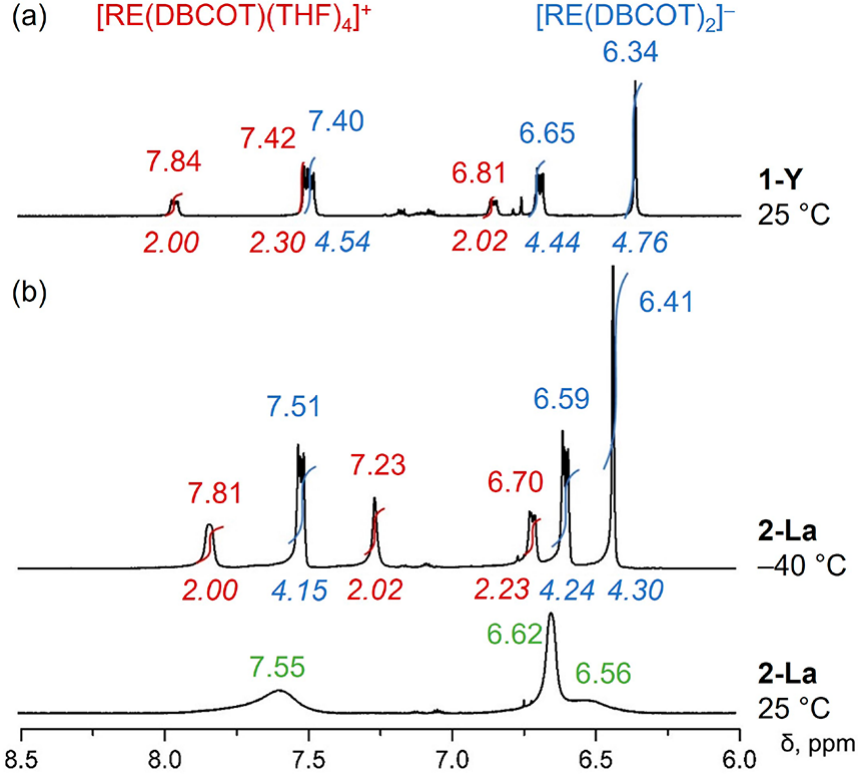
^1^H NMR spectra of (a) **1-Y** at 25 °C and (b) **2-La** at +25 and −40 °C in THF-*d*_8_, aromatic regions with chemical shifts and integrations.

The solution behavior of these types of products has been investigated with NMR spectroscopy using the diamagnetic **1-Y** and **2-La** analogues. In the ^1^H NMR spectrum of **1-Y**, two sets of signals can be identified with a ratio of 1:2 for DBCOT^2–^ anions, which corresponds to the cationic and anionic moieties. Signals of the cationic half-sandwich, [Y(DBCOT)(THF)_4_]^+^, appear as multiplets at 6.81 and 7.84 ppm and a singlet at 7.42 ppm (Figure 5a).

Compared to K_2_DBCOT, the proton signals in the cationic part of **1-Y** show a similar pattern but are overall more deshielded, covering a narrower range (6.81–7.84 ppm vs 6.19–7.87 ppm in K_2_DBCOT)^28^ due to direct Y(III) coordination. For the anionic sandwich [Y(DBCOT)_2_]^−^, the corresponding signals appear as multiplets at 6.65 and 7.40 ppm and a singlet at 6.34 ppm, with the values being comparable to those reported for [K(DME)_2_Y(DME)(DBCOT)_2_].^29^ The proton signals of the terminal six-membered rings show smaller variations (6.65–7.40 ppm vs 7.01–7.10 ppm in K_2_DBCOT), but those of the central eight-membered rings are much more shielded (6.34 ppm vs 7.17 ppm in K_2_DBCOT). Notably, the ^1^H NMR spectrum of **2-La** shows three broad peaks, corresponding to the three types of protons of DBCOT^2–^ in different environments (Figure 5b), thus indicating that the cationic and anionic parts weakly interact in solution at room temperature. Upon the temperature decrease to −20 °C (Figure S5), the proton signals start to resolve and appear as two sets of peaks for the DBCOT^2–^ anions. Because a further temperature decrease causes only minor changes in the chemical shifts, the spectrum at −40 °C is selected for comparison. Compared to **1-Y**, proton signals corresponding to [La(DBCOT)(THF)_4_]^+^ are slightly upfield-shifted, appearing as broad signals at 6.70, 7.23, and 7.81 ppm, respectively. For the [La(DBCOT)_2_]^−^ sandwich, the corresponding signals are slightly downfield-shifted and appear as multiplets at 6.59 and 7.51 ppm, along with a singlet at 6.41 ppm. This indicates an increased electron density in the center of the DBCOT core in both **1-Y** and **2-La**, similar to the observation in the rare-earth COT-based triple deckers.^9^

### Magnetic Properties

**Figure 6:**
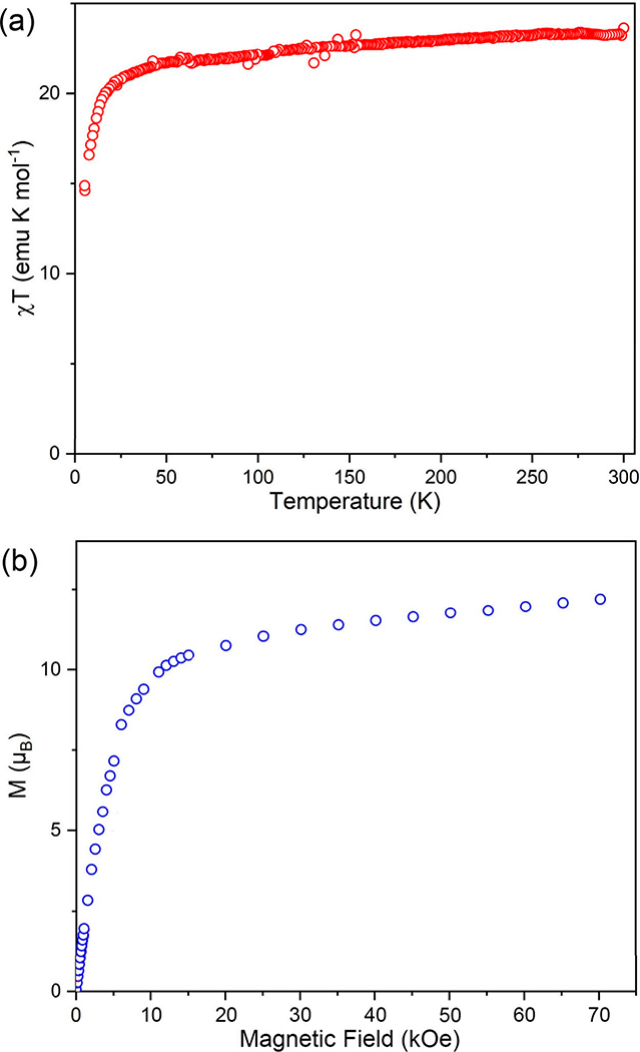
Magnetic properties of **5-Dy**: temperature dependence of χ*T* measured under a dc magnetic field of 1000 Oe (a) and field-dependent magnetization measured at 1.8 K (b).

To compare to the previously reported [K([2.2.2]cryptand)][Cp^tet^_2_Dy(DBCOT)]^30^ and to evaluate the effect of ligand replacement, the molar magnetic susceptibility (χ) was measured on a powder sample of **5-Dy** sealed in an NMR tube. At room temperature, the value of the χ*T* product is 23.24 emu·K/mol, somewhat lower than the theoretical value of 28.33 emu·K/mol expected for a compound with two Dy^3+^ centers in the absence of magnetic exchange coupling (this discrepancy might be due to the presence of a minor diamagnetic impurity or slight inaccuracy in the mass measurement associated with handling the sample under a rigorously anaerobic environment). The χ*T* product remains relatively constant until about 50 K (Figure 6a), at which point it decreases quickly as the temperature is lowered, reaching 14.80 emu·K/mol at 1.8 K. The field-dependent magnetization measured at 1.8 K saturated to a value of 11.8 μ_B_ at 70 kOe (Figure 6b), which is substantially lower than the 20.0 μ_B_ expected for the two Dy^3+^ ions. The rapid decrease in the χ*T* product below 50 K and the lack of maximum magnetization at 1.8 K and 70 kOe can be attributed to crystal-field-splitting effects.

**Figure 7:**
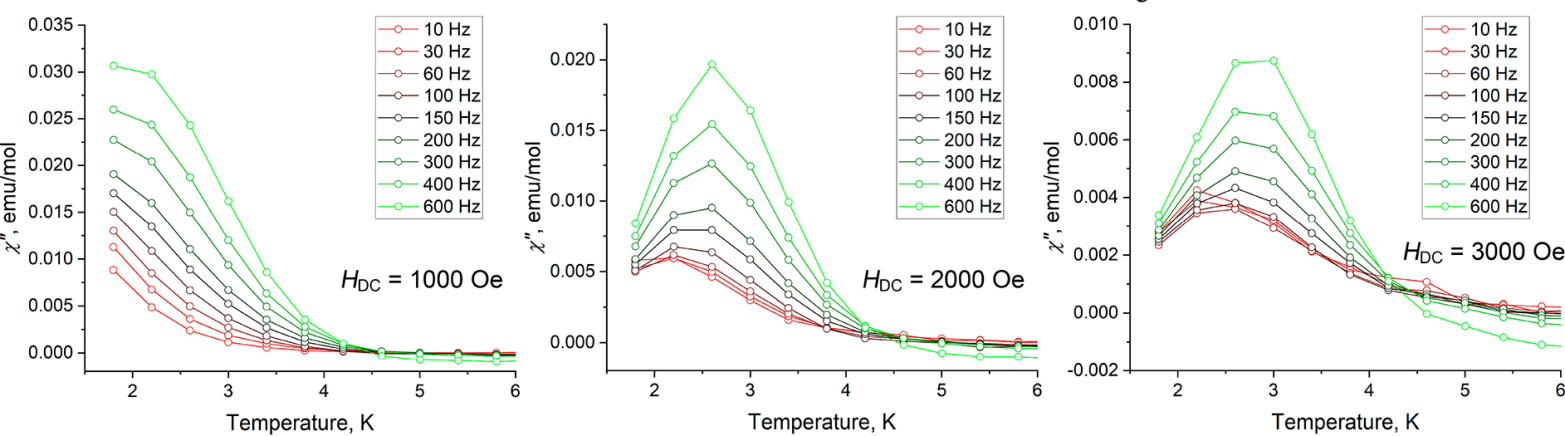
Temperature dependence of the out-of-phase ac magnetic susceptibility of **7-Dy/Y** measured under variable bias dc magnetic fields (*H*_dc_).

To investigate whether **5-Dy** exhibits SMM properties, alternating-current (ac) magnetic susceptibility was measured in the 1.8–9.0 K range. Measurements on the pure sample of **5-Dy** did not lead to the observation of a clear out-of-phase ac susceptibility (χ″) signal characteristic of SMMs, but a signature of such a signal became evident at the lowest accessible temperature of 1.8 K (Figure S18a). To decrease the impact of dipolar coupling between the paramagnetic Dy^3+^ centers on the relaxation of magnetization, we prepared a solid solution of **5-Dy** with its diamagnetic Y(III) analogue, i.e., [Dy_0.2_Y_0.8_(DBCOT)(THF)_4_][Dy_0.2_Y_0.8_(DBCOT)_2_] (**7-Dy/Y**). This sample revealed a clear onset of peaks in the temperature dependence of χ″ measured in a zero direct-current (dc) magnetic field and at variable frequencies of the ac magnetic field, despite the substantial noise in the data (Figure S18b). Next, the ac susceptibility measurements were repeated under various applied dc fields, which immediately revealed frequency-dependent maxima in the χ″ versus *T* dependence that were shifting to higher temperatures with increasing magnetic field (Figure 7). The appearance of these peaks indicates that the applied dc bias field suppresses the relaxation of magnetization by quantum-tunneling pathways, as is commonly observed for SMMs.

**Figure 8:**
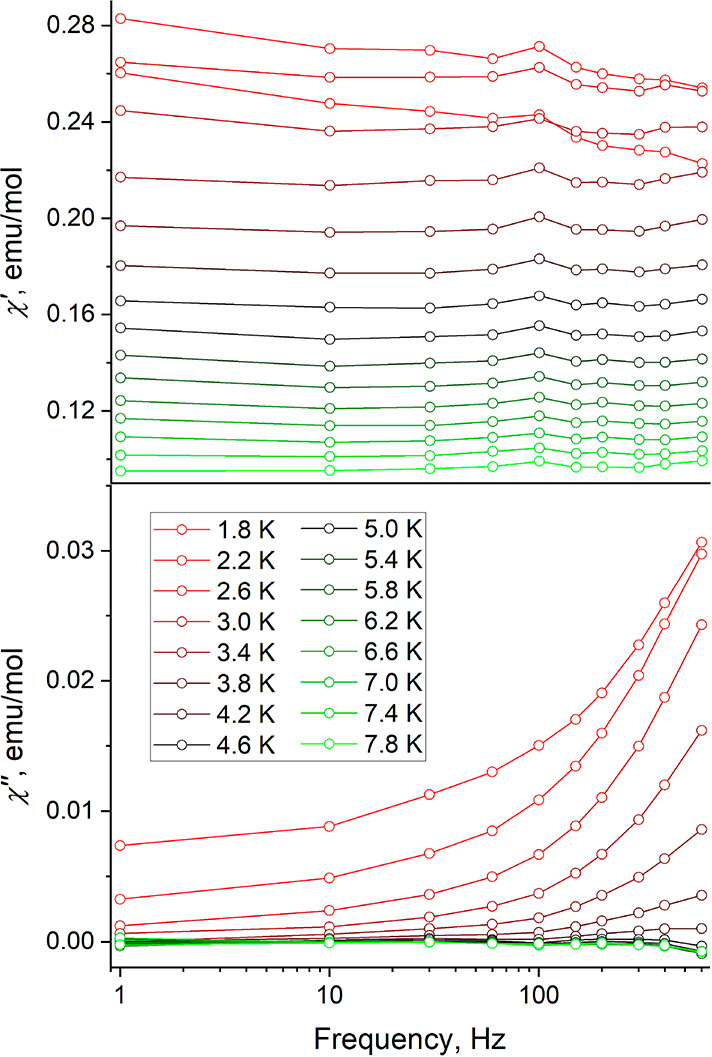
Frequency dependence of the in-phase (χ′) and out-of-phase (χ″) components of the ac susceptibility of **7-Dy/Y** measured at variable temperature and under an applied dc field of 1000 Oe.

The SMM behavior of this complex is substantially weaker than that of previously reported related compounds, [DyKCa(COT)_3_(THF)_3_]^34^ and [K(crypt-222)][Cp^tet^_2_Dy(η^2^-DBCOT)].^30^ Indeed, in both previous cases, the out-of-phase peaks were observed at higher temperatures and even for the ac magnetic susceptibility measured under zero dc field. The dissipation of the in-phase ac susceptibility (χ′) in favor of the out-of-phase component (χ″) also was evident at ac field frequencies exceeding 100 Hz, while in the present case, such dissipation is much weaker (Figure 8), although the onset of the peak in the frequency dependence of χ″ is observed, as expected for a SMM. The deterioration of the SMM properties in the current Dy complex could stem from the lower rigidity of the ligand environment. Both the presence of peripheral rings in DBCOT and the coordination of flexible THF molecules provide multiple energetically accessible vibrational pathways for the relaxation of magnetization^35^ (the Orbach and Raman relaxation processes). Thus, even when the applied dc field suppresses quantum tunneling, the SMM behavior remains rather weak. This situation contrasts with the presence of more rigid COT and Cp^tet^ ligands in [DyKCa(COT)_3_(THF)_3_]^34^ and [K(crypt-222)][Cp^tet^_2_Dy(η^2^-DBCOT)],^30^ respectively.

### Theoretical Computation

To better understand this phenomenon, density functional theory (DFT) and *ab initio* calculations were performed for **5-Dy**, with the *ORCA* electronic structure suite.^36−38^ Throughout the following section, the anionic [Dy(DBCOT)_2_]^−^ system is labeled as **5-a**, and the [Dy(DBCOT)(THF)_4_]^+^ cation is labeled as **5-c**. The calculated nephelauxetic reductions and relativistic nephelauxetic reductions for the systems show that the reductions observed for **5-a** are larger than those found for **5-c**, which highlights stronger covalent interactions between the DBCOT^2–^ ligands compared to THF (Figure S19). Notably, this feature is greatly diminished when inspecting the corresponding nephelauxetic reductions based on the complete-active-space self-consistent-field (CASSCF) results (Figure S20). This may indicate that dynamic correlation plays an important role in covalent bonding between the Dy^3+^ cation and DBCOT^2–^/THF ligands.

Both the DFT and CASSCF results suggest that the strongest interactions are between the DBCOT π system and the Dy 5d shell (Table 2). Average Dy–C_COT_ Wiberg bond orders (WBOs) of 0.16 and 0.14 are observed for **5-a** and **5-c**, respectively, as calculated at the DFT level of theory. Compared to Ln centers entrapped between COT^2–^, the WBOs for the anionic sandwich **5-a** appear to be lower, illustrating the loosened bonding between Dy and DBCOT^2–^. The 5d populations in these systems correlate with the donor–acceptor stabilizations between the DBCOT π system and the 5d shell, as determined through second-order perturbation analysis of the Fock matrix on a natural-bond-order basis.

**Table 2:** Natural Population Analysis as Applied to the DFT and SA-CASSCF(*n*,7) Results

		**5-a**	**5-c**
CASSCF	charge	1.55	2.03
	4f	9.01	9.01
	5d	1.25	0.71
	6s	0.11	0.12
DFT	charge	1.36	1.82
	4f	9.12	9.10
	5d	1.32	0.79
	6s	0.12	0.13

**Figure 9:**
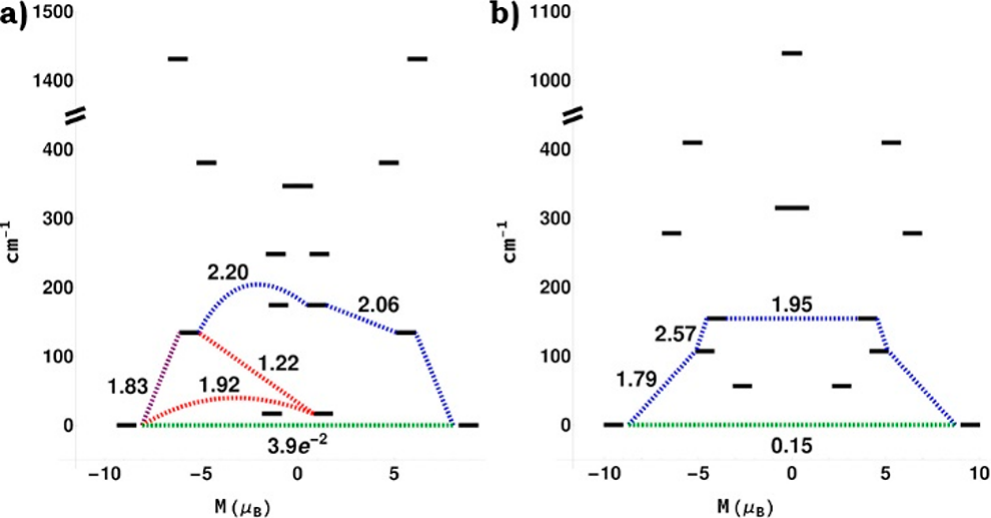
Blocking diagram from NEVPT2(9,7)/QDPT/SINGLE_ANISO calculations on (a) **5-a** and (b) **5-c**. Blue lines correspond to the most likely relaxation pathways, green lines correspond to quantum tunneling of magnetization, and red/purple lines highlight competing pathways in part a.

The NEVPT2(n,7)/QDPT/SINGLE_ANISO^39−41^ module was then used to study the ground-state multiplets of the target systems (see the computational methods in the Supporting Information). The blocking diagrams for **5-a** (Figure 9a) show that there are multiple competing relaxation pathways for this system, consistent with the experimental results. This specific behavior can be attributed to the reduced symmetry present in these systems, which strongly mixes different *M*_*J*_ states.

## Conclusions

In summary, a series of new rare-earth complexes [RE(DBCOT)(THF)_4_][RE(DBCOT)_2_] (RE = Y, La, Gd, Tb, Dy, and Er) with the doubly reduced DBCOT were synthesized and fully characterized. Single-crystal X-ray diffraction confirmed that all complexes are composed of the monoanionic [RE(DBCOT)_2_]^−^ sandwich, which is weakly interacting with a cationic counterpart, [RE(DBCOT)(THF)_4_]^+^. Structural analysis revealed that a RE(III) ion is sandwiched between the central eight-membered rings in an η^8^ fashion with RE–COT_centroid_ distances of 1.855(5)–2.084(8) Å. The two elongated π decks are almost parallel (2.0–3.1°) but slightly bent toward the metal center (3.9–10.1°) with a rotation angle of 35.9–37.6°. The first homoleptic sandwich-type product of Dy(III) with the η^8^-coordinated doubly reduced DBCOT^2–^ anions was subjected to magnetic property measurements, which revealed SMM behavior with a rather small magnetization reversal barrier. The inferior SMM properties of this Dy-containing complex compared to the previously reported SMM behavior of the related [DyKCa(COT)_3_(THF)_3_]^34^ and [K(crypt-222)][Cp^tet^_2_Dy(η^2^-DBCOT)]^30^ can be attributed to the more accessible vibrational pathways for the relaxation of magnetization in the present case, which is further supported by the DFT and *ab initio* levels of theory, as shown in Figure 9. Theoretical analysis of the ground states of two different Dy(III) ions and blocking diagrams revealed a multifaceted relaxion mechanism, in full agreement with the hypothesis conjectured from the experimental observations.

## Experimental Section

### Materials and Methods

All manipulations were carried out using break-and-seal and glovebox techniques under an atmosphere of argon.^42^ Tetrahydrofuran (THF) and hexanes (Sigma-Aldrich) were dried over Na/benzophenone and distilled prior to use. THF-*d*_8_ (≥99.5 atom % D, Sigma-Aldrich) was dried over a NaK_2_ alloy and vacuum-transferred. DBCOT (97%) was purchased from Tokyo Chemical Industry and sublimed under reduced pressure in a 10 cm ampule at 78 °C over 4 days. Potassium metal (98%) was purchased from Sigma-Aldrich and used as received. YI_3_ (99.9%), LaI_3_ (99.9%), GdI_3_ (99.99%), TbI_3_ (99.99%), DyI_3_ (99.99%), and ErI_3_ (99.9%) were purchased from Alfa Aesar and used as received. K_2_DBCOT was prepared according to literature procedures and stored in a glovebox.^28^ All crystals were obtained by slow solvent evaporation in sealed L-shaped glass ampules (Figure S10). The air sensitivity of products **1**–**6**, along with the presence of loosely bound THF molecules, prevented obtainment of elemental analysis data. The attenuated-total-reflection infrared (ATR-IR) spectra for **1**–**5** were recorded on a PerkinElmer Spectrum 100 FT-IR spectrometer; for **6-Er**, a Shimadzu IRTracer-100 FT-IR QATR10 spectrometer (a single reflection ATR accessory) was used. The UV–vis absorption spectra were recorded on Thermo Scientific Evolution 201 and Shimadzu UV-2600i UV–vis spectrophotometers. The ^1^H NMR spectra were recorded on a Bruker Ascend-500 spectrometer (500 MHz for ^1^H). Chemical shifts (δ) are reported in parts per million (ppm) and referenced to the resonances of the corresponding solvent used. The low-temperature NMR experiment was controlled by a Cryo Diffusion cryogenic tank probe, and liquid N_2_ was used as a cooling source.

#### [Y(DBCOT)(THF)_4_][Y(DBCOT)_2_] (**1-Y**)

A THF (1.0 mL) solution of K_2_DBCOT (12.1 mg, 0.043 mmol) was added to a customized glass system containing a slurry of YI_3_ (13.0 mg, 0.028 mmol) in THF (1.0 mL). The reaction mixture was stirred at 25 °C under argon for 48 h. The initial red color (K_2_DBCOT) changed to orange in 10 h. The mixture was filtered after 48 h, and the orange filtrate was sealed in an L-shaped ampule under reduced pressure. The L-shaped ampule was placed over a hot sand bath (110 °C) for slow solvent evaporation. After 10 days, orange block-shaped crystals appeared in the ampule. Yield: 7.6 mg, 50%. IR: 1144, 1011, 995, 851, 831, 808, 768, 735, 710, 659, 603 cm^–1^. UV–vis (THF): λ_max_ 306 (sh), 320 (sh) nm. ^1^H NMR (THF-*d*_8_, 25 °C): δ 1.77 (16H, [Y(DBCOT)(THF)_4_]^+^), 3.62 (16H, [Y(DBCOT)(THF)_4_]^+^), 6.34 (8H, [Y(DBCOT)_2_]^−^), 6.65 (8H, [Y(DBCOT)_2_]^−^), 6.81 (4H, [Y(DBCOT)(THF)_4_]^+^), 7.40 (8H, [Y(DBCOT)_2_]^−^), 7.42 (4H, [Y(DBCOT)(THF)_4_]^+^), 7.84 (4H, [Y(DBCOT)(THF)_4_]^+^).

#### [La(DBCOT)(THF)_4_][La(DBCOT)_2_] (**2-La**)

A THF (1.0 mL) solution of K_2_DBCOT (12.0 mg, 0.042 mmol) was added to a customized glass system containing a slurry of LaI_3_ (14.6 mg, 0.028 mmol) in THF (1.0 mL). The reaction mixture was stirred at 25 °C under argon for 48 h. The initial red color (K_2_DBCOT) changed to orange in 10 h. The mixture was filtered after 48 h, and the orange filtrate was sealed in an L-shaped ampule under reduced pressure. The L-shaped ampule was placed over a hot sand bath (110 °C) for slow solvent evaporation. After 10 days, orange block-shaped crystals appeared in the ampule. Yield: 7.6 mg, 50%. IR: 1145, 1007, 852, 831, 798, 765, 721, 654, 603 cm^–1^. UV–vis (THF): λ_max_ 308, 322 nm. ^1^H NMR (THF-*d*_8_, 25 °C): δ 1.79 (16H, [La(DBCOT)(THF)_4_]^+^), 3.62 (16H, [La(DBCOT)(THF)_4_]^+^), 6.41 (8H, [La(DBCOT)_2_]^−^), 6.59 (8H, [La(DBCOT)_2_]^−^), 6.70 (4H, [La(DBCOT)(THF)_4_]^+^), 7.23 (8H, [La(DBCOT)(THF)_4_]^+^), 7.51 (4H, [La(DBCOT)_2_]^−^), 7.81 (4H, [La(DBCOT)(THF)_4_]^+^).

#### [Gd(DBCOT)(THF)_4_][Gd(DBCOT)_2_] (**3-Gd**)

A THF (1.0 mL) solution of K_2_DBCOT (6.9 mg, 0.024 mmol) was added to a customized glass system containing a slurry of GdI_3_ (8.6 mg, 0.016 mmol) in THF (1.0 mL). The reaction mixture was stirred at 25 °C under argon for 48 h. The initial red color (K_2_DBCOT) changed to dark brown in 5 h. The mixture was filtered after 48 h, and the dark-brown filtrate was sealed in an L-shaped ampule under reduced pressure. The L-shaped ampule was placed over a hot sand bath (140 °C) for slow solvent evaporation. After 14 days, dark-brown block-shaped crystals appeared in the ampule. Yield: 8.5 mg, 50%. IR: 1145, 1013, 998, 853, 831, 806, 766, 735, 710, 660, 604 cm^–1^. UV–vis (THF): λ_max_ 308, 323 nm.

#### [Tb(DBCOT)(THF)_4_][Tb(DBCOT)_2_] (**4-Tb**)

A THF (1.0 mL) solution of K_2_DBCOT (7.0 mg, 0.025 mmol) was added to a customized glass system containing a slurry of TbI_3_ (8.8 mg, 0.016 mmol) in THF (1.0 mL). The reaction mixture was stirred at 25 °C under argon for 48 h. The initial red color (K_2_DBCOT) changed to dark orange in 5 h. The mixture was filtered after 48 h, and the dark-orange filtrate was sealed in an L-shaped ampule under reduced pressure. The L-shaped ampule was placed over a hot sand bath (140 °C) for slow solvent evaporation. After 14 days, dark-brown block-shaped crystals appeared in the ampule. Yield: 8.4 mg, 50%. IR: 1144, 1011, 999, 852, 831, 809, 767, 722, 710, 659, 604 cm^–1^. UV–vis (THF): λ_max_ 307, 322 nm.

#### [Dy(DBCOT)(THF)_4_][Dy(DBCOT)_2_] (**5-Dy**)

A THF (1.0 mL) solution of K_2_DBCOT (6.9 mg, 0.024 mmol) was added to a customized glass system containing a slurry of DyI_3_ (8.6 mg, 0.016 mmol) in THF (1.0 mL). The reaction mixture was stirred at 25 °C under argon for 48 h. The initial red color (K_2_DBCOT) changed to brown in 5 h. The mixture was filtered after 48 h, and the brown filtrate was sealed in an L-shaped ampule under reduced pressure. The L-shaped ampule was placed over a hot sand bath (140 °C) for slow solvent evaporation. After 14 days, dark-brown block-shaped crystals appeared in the ampule. Yield: 7.1 mg, 50%. IR: 1144, 1010, 997, 851, 830, 810, 766, 722, 710, 660, 604 cm^–1^. UV–vis (THF): λ_max_ 307, 322 nm.

#### [Er(DBCOT)(THF)_4_][Er(DBCOT)_2_] (**6-Er**)

A THF (1.0 mL) solution of K_2_DBCOT (8.0 mg, 0.028 mmol) was added to a customized glass system containing a slurry of ErI_3_ (10.4 mg, 0.019 mmol) in THF (1.0 mL). The reaction mixture was stirred at 25 °C under argon for 48 h. The initial red color (K_2_DBCOT) changed to brown in 4 h. The mixture was filtered after 48 h, and the brown filtrate was sealed in an L-shaped ampule under reduced pressure. The L-shaped ampule was placed over a hot sand bath (140 °C) for slow solvent evaporation. After 12 days, dark-brown block-shaped crystals appeared in the ampule. Yield: 8.3 mg, 59%. IR: 1168, 1143, 1007, 993, 847, 827, 810, 764, 755, 736, 709, 659, 603 cm^–1^. UV–vis (THF): λ_max_ 306, 378, 398, 423 nm.

### Crystal Structure Solution and Refinement

Data collection of **1-Y**, **2-La**, **3-Gd**, **4-Tb**, and **5-Dy** was performed on a Bruker VENTURE system equipped with a PHOTON 100 CMOS detector, a Mo-target fine-focus X-ray source (λ = 0.71073 Å), and a graphite monochromator. All data were collected at a 100(2) K crystal temperature (Oxford Cryosystems CRYOSTREAM 700), 50 kV, and 30 mA with an appropriate 0.5° ω scan strategy. All data reduction and integration were performed with *SAINT* (version 8.38A).^43^ All five data were corrected for absorption effects using the empirical methods, as implemented in *SADABS* (version 2016/2).^44^ Data collection of **6-Er** was performed at 100.00(10) K on a Rigaku XtaLAB Synergy-S X-ray diffractometer equipped with a HyPix-6000HE hybrid-photon-counting detector and microfocus Cu Kα radiation (λ = 1.54178 Å). A data collection strategy to ensure completeness and the desired redundancy was determined using *CrysAlisPro*.^45^ Data processing was performed using *CrysAlisPro*.^45^ Empirical absorption correction was applied using the *SCALE3 ABSPACK* scaling algorithm.^46^ All structures were solved by *SHELXT* (version 2018/2)^47^ and refined by full-matrix least-squares procedures using the *SHELXL* program (version 2019/2 for **2-La** and **6-Er** and version 2018/3 for the rest)^48^ through the *OLEX2*(49) graphical interface. All non-H atoms, including those in disordered parts, were refined anisotropically. All H atoms were included at calculated positions and refined as riders, with *U*_iso_(H) = 1.2*U*_eq_(C). In **2-La**, the sandwiched La(III) ion and one THF molecule were found to be disordered. This La(III) ion was found to be disordered over two positions, which were constrained to have identical anisotropic displacement parameters. The La(III) ions at these two positions were found to have different coordination with the DBCOT dianions, specifically η^8^ and η^5^ coordination (Figure S17). Their ratio was refined to 0.8942(2):0.1058(12). In **3-Gd** and **4-Tb**, two THF molecules were found to be disordered in each structure. All disordered THF molecules were modeled with two orientations with their relative occupancies refined. The geometries of the disordered parts were restrained to be similar. The anisotropic displacement parameters of the disordered molecules in the direction of the bonds were restrained to be equal with a standard uncertainty of 0.004 Å^2^. They were also restrained to have the same *U*_*ij*_ components, with a standard uncertainty of 0.01 Å^2^. In **5-Dy**, in order to make all C atoms’ anisotropic displacement parameters reasonable, the anisotropic displacement parameters of the DBCOT dianions in the direction of the bonds were restrained to be equal with a standard uncertainty of 0.004 Å^2^. They were also restrained to have the same *U*_*ij*_ components, with a standard uncertainty of 0.01 Å^2^. Besides, **1-Y**, **2-La**, **4-Tb**, **5-Dy**, and **6-Er** were refined as inversion twins with BASF values refined to 0.445(3), 0.466(25), 0.348(10), 0.403(11), and 0.485(7), respectively. These five structures were examined by *PLATON*,^50^ and no additional symmetry was found. Crystallographic data and details of the data collection and structure refinement are listed in Table S5. The ORTEP drawings for **1**–**6** are shown in the Supporting Information.

### Magnetic Measurements

The powder samples of **5-Dy** and **7-Dy/Y** were studied using a magnetic property measurement system (MPMS-3, Quantum Design) equipped with a superconducting quantum interference device (SQUID). The samples were sealed in standard NMR tubes, which were mounted inside a plastic straw and attached to the sample transport rod. The dc magnetic susceptibility was measured in an applied field of 1000 Oe in the 1.8–300 K temperature range. The ac magnetic susceptibility was measured in the 1.8–7.8 K temperature range, with 0.4 K steps, in zero magnetic field and then under variable applied dc magnetic field. The ac frequency was varied from 1 to 600 Hz. The data were corrected for a diamagnetic contribution from the sample holder and for the intrinsic diamagnetism of the samples.

## Supporting Information Available

X-ray structural details, UV–vis and ATR-IR spectroscopic data, magnetic measurements, and computational details (PDF)

## Dedication

Dedicated to the memory of Prof. Alexander V. Nemukhin, a great mentor and friend.
