# Supplementary material for: Homoleptic Rare-Earth-Metal Sandwiches with Dibenzo[a,e]cyclooctatetraene Dianions
Source: Inorg Chem. 2024 Feb 20;63(21):9579–87. doi: 10.1021/acs.inorgchem.3c04249 (PMC11134502; doi:10.1021/acs.inorgchem.3c04249)
Supplement: Supplementary file 1 — ic3c04249_si_001.pdf [file ic3c04249_si_001.pdf]

Supporting Information

Homoleptic Rare-Earth Metal Sandwiches with Dibenzo[*a*,*e*]cyclooctatetraene Dianions

Yikun Zhu,[a] James Mahoney,[a] Aaron J. Babson,[a] Zheng Zhou,[a,b] Zheng Wei,[a] Miguel Gakiya-Teruya,[c] James McNeely,[d] Andrey Yu. Rogachev,[e,*] Michael Shatruk,[c,*] and Marina A. Petrukhina[a,*]

[a] Department of Chemistry, University at Albany, State University of New York, Albany, NY 12222, USA

[b] Interdisciplinary Materials Research Center, School of Materials Science and Engineering, Tongji University, Shanghai 201804, China

[c] Department of Chemistry and Biochemistry, Florida State University, Tallahassee, FL 32306, USA

[d] Department of Chemistry, Boston University, Boston, MA 02215, USA

[e] Department of Chemistry, Illinois Institute of Technology, Chicago, IL 60616, USA

[*] E-mail: mpetrukhina@albany.edu

I. UV-vis Spectroscopic Investigation 1

II. ATR-IR Spectroscopic Investigation 2

III. NMR Spectroscopic Investigation 3

IV. X-ray Powder Diffraction Results 4

V. Crystal Structure Solution and Refinement 8

VI. Magnetic Properties 13

VII. Computational Methods 14

VIII. References 18

I. UV-vis Spectroscopic Investigation

Sample preparation: THF (3.0 mL) was added to a glass ampule (O.D. 12 mm) containing **1-Y**, **2-La**, **3-Gd**, **4-Tb**, **5-Dy**, or **6-Er** (0.2 mg). The ampule was sealed under argon, and UV-vis absorption spectrum was collected at 25 °C.

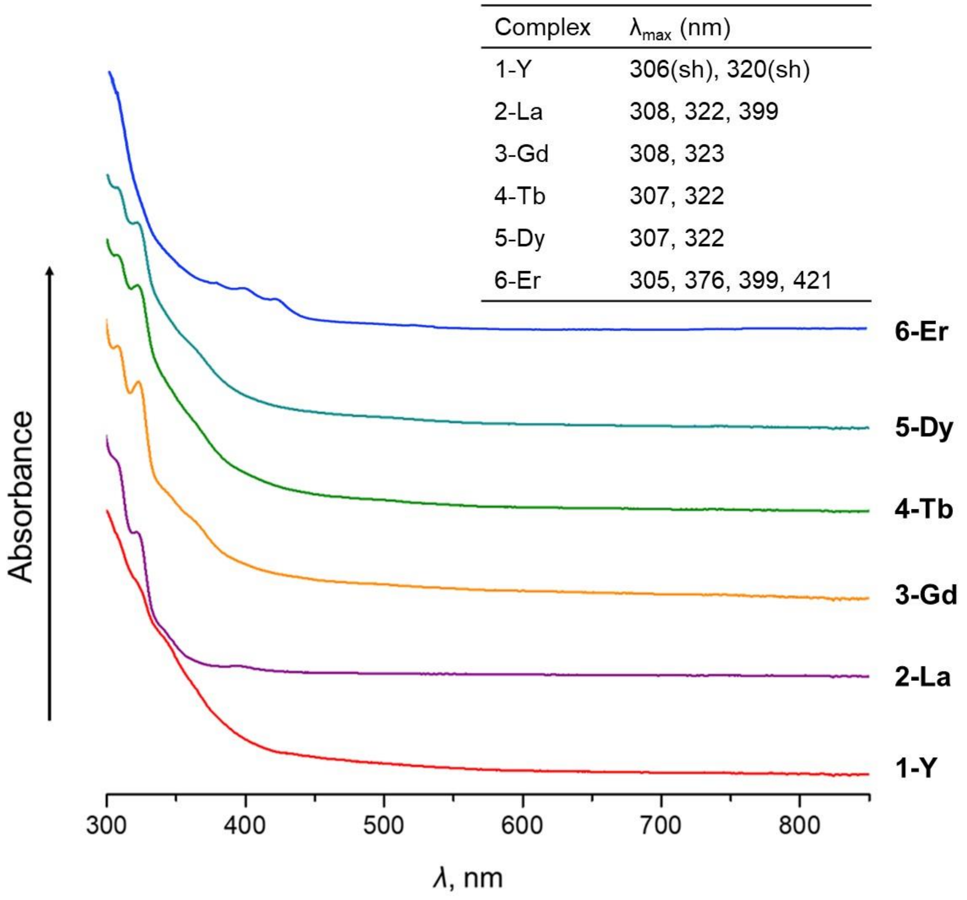

Complex	λ_{max} (nm)
1-Y	306(sh), 320(sh)
2-La	308, 322, 399
3-Gd	308, 323
4-Tb	307, 322
5-Dy	307, 322
6-Er	305, 376, 399, 421

Figure S1. UV-vis spectra of **1-Y**, **2-La**, **3-Gd**, **4-Tb**, **5-Dy**, and **6-Er** in THF.

II. ATR-IR Spectroscopic Investigation

Sample preparation: 0.5 mg of orange/brown crystalline products of **1-Y**, **2-La**, **3-Gd**, **4-Tb**, **5-Dy**, or **6-Er** was covered in mineral oil. The sample was only scanned twice due to its moisture and air sensitivity.

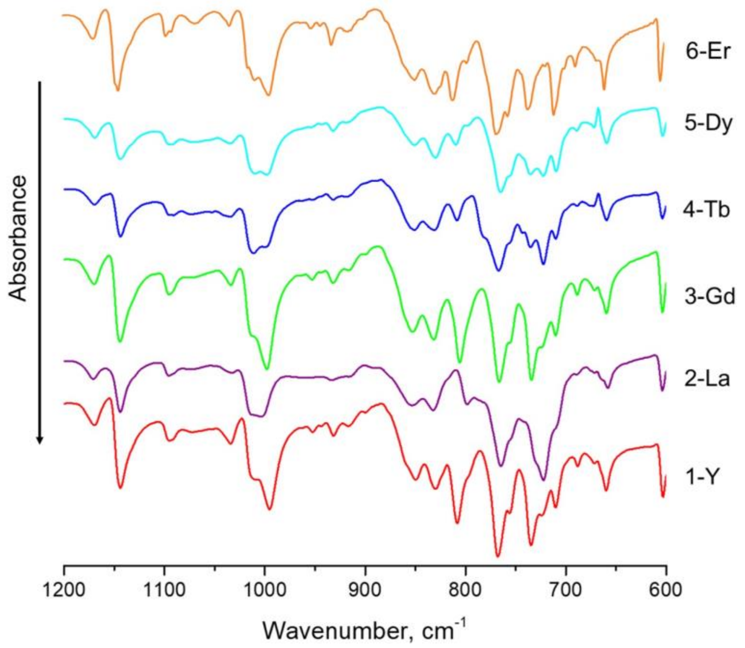

Figure S2. ATR-IR spectra of **1-Y**, **2-La**, **3-Gd**, **4-Tb**, **5-Dy**, and **6-Er**, fingerprint region.

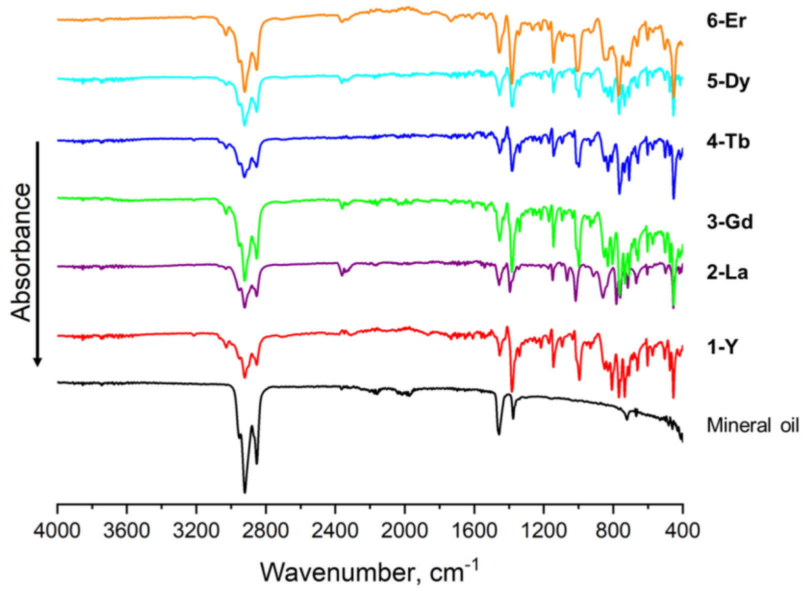

Figure S3. ATR-IR spectra of **1-Y**, **2-La**, **3-Gd**, **4-Tb**, **5-Dy**, and **6-Er**, along with mineral oil (reference), full region.

III. NMR Spectroscopic Investigation

Sample preparation: Crystals of **1-Y** (2.0 mg) were washed several times with hexanes, dried *in-vacuo*, and dissolved in THF-d_8 (0.70 mL). The resulting solution was transferred to an NMR tube that was sealed under argon.

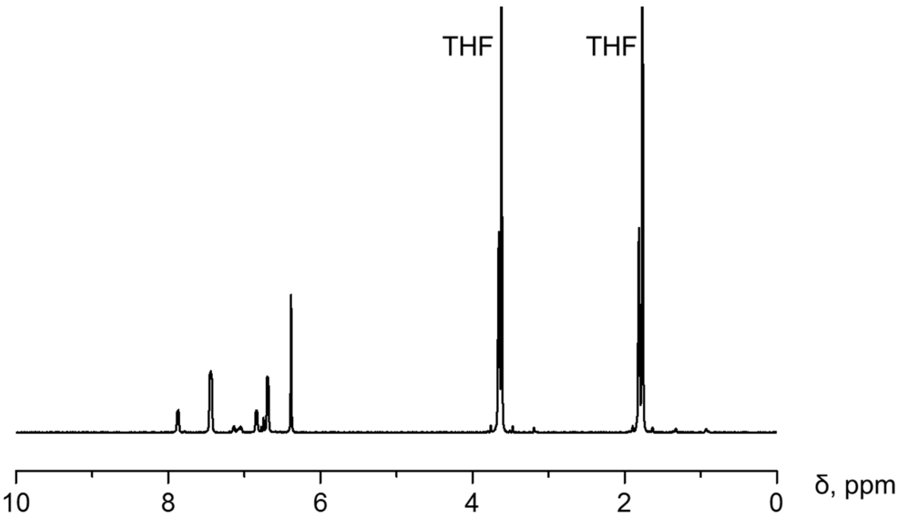

Figure S4. ^{1}H NMR spectrum of **1-Y** in THF-d_8 at 25 °C.

Sample preparation: Crystals of **2-La** (2.0 mg) were washed several times with hexanes, dried *in-vacuo*, and dissolved in THF-d_8 (0.70 mL). The resulting solution was transferred to an NMR tube that was sealed under argon.

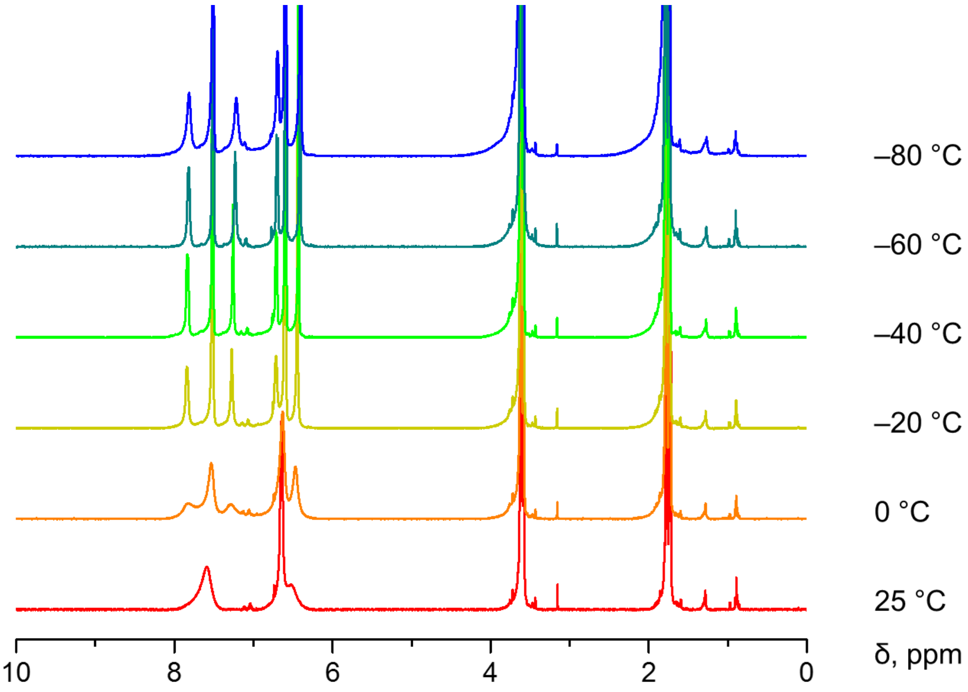

Figure S5. Variable-temperature ^{1}H NMR spectra of **2-La** in THF-d_8.

IV. X-ray Powder Diffraction Results

The X-ray powder diffraction data were collected on a Bruker D8 Venture single crystal diffractometer (Cu Kα INCOATEC IμS microfocus source, focusing multilayer mirrors monochromator, Photon 100 detector, 100(2) K). The crystalline samples under investigation were ground under Ar in the glove-box and mounted on a MiTeGen 10 μm MicroMount protected by a nitrogen gas flow. Le Bail fit for powder diffraction patterns was performed using TOPAS, version 4 software package (Bruker AXS, 2006).

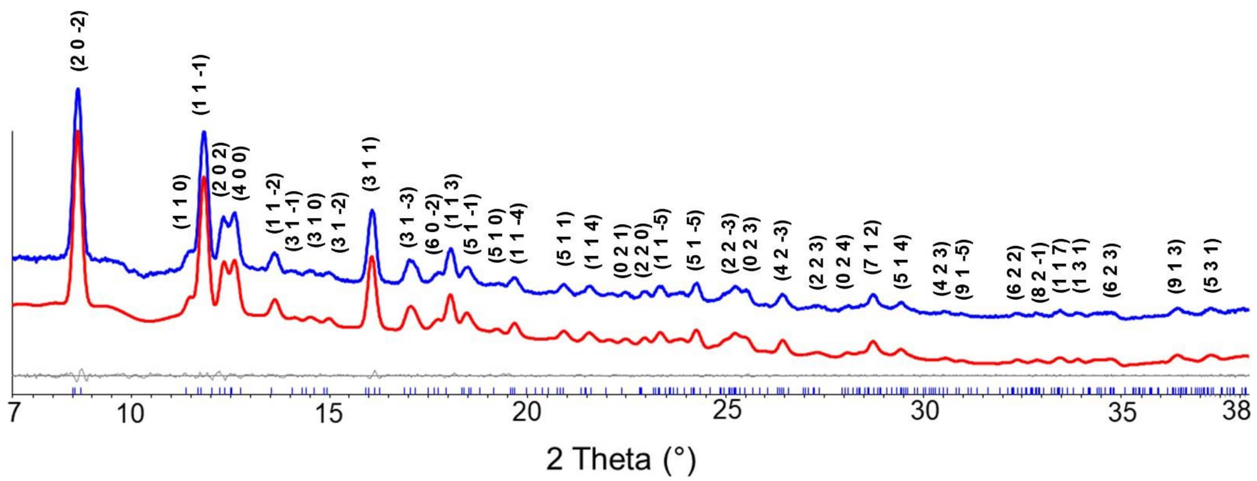

Figure S6. X-ray powder diffraction pattern of **3-Gd** and Le Bail fit. The blue and red lines are experimental and calculated patterns respectively. The grey line is the difference curve with theoretical positions shown at the bottom in blue.

Table S1. Comparison of unit cell parameters for **3-Gd** from single crystal data and the Le Bail fit.

	Single crystal data (100 K)	Le Bail fit data (100 K)
Space Group	*C*2	
a (Å)	30.372(2)	30.3686(18)
b (Å)	8.0988(6)	8.0979(6)
c (Å)	22.3088(16)	22.3055(16)
α (°)	90.00	90.00
β (°)	111.070(1)	111.062(3)
γ (°)	90.00	90.00
V (Å^3)	5120.5(6)	5118.9(6)

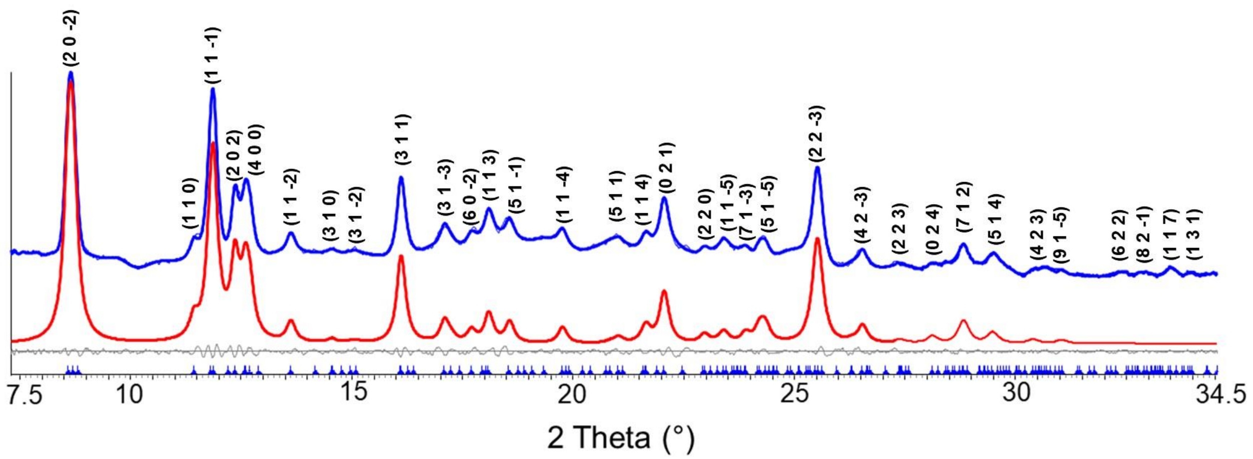

Figure S7. X-ray powder diffraction pattern of **4-Tb** and Le Bail fit. The blue and red lines are experimental and calculated patterns, respectively. The grey line is the difference curve with theoretical positions shown at the bottom in blue arrows.

Table S2. Comparison of unit cell parameters for **4-Tb** from single crystal data and the Le Bail fit.

	Single crystal data (100 K)	Le Bail fit data (100 K)
Space Group	*C*2	
a (Å)	30.337(3)	30.318(9)
b (Å)	8.0750(7)	8.0738(9)
c (Å)	22.305(2)	22.320(8)
α (°)	90.00	90.00
β (°)	111.2380(10)	111.2395(11)
γ (°)	90.00	90.00
V (Å^3)	5093.0(8)	5092.4(9)

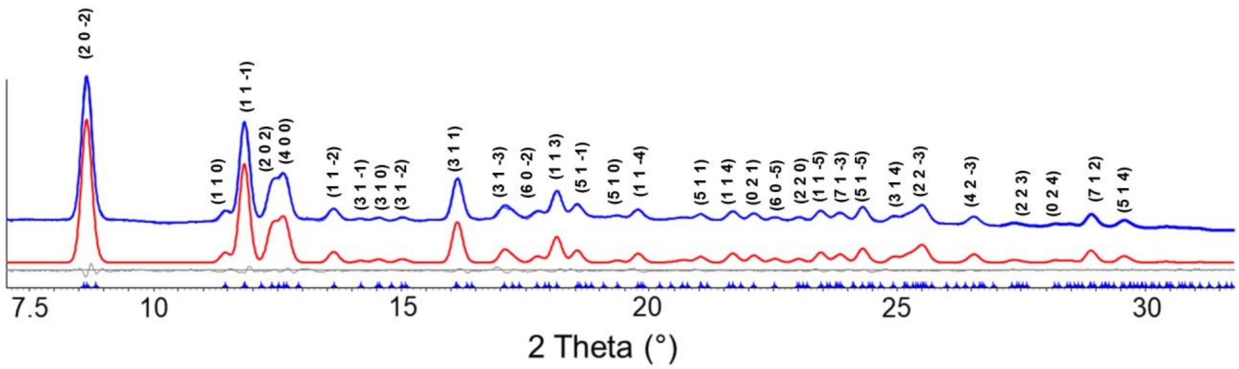

Figure S8. X-ray powder diffraction pattern of **5-Dy** and Le Bail fit. The blue and red lines are experimental and calculated patterns, respectively. The grey line is the difference curve with theoretical positions shown at the bottom in blue arrows.

Table S3. Comparison of unit cell parameters for **5-Dy** from single crystal data and the Le Bail fit.

	Single crystal data (100 K)	Le Bail fit data (100 K)
Space Group	*C*2	
a (Å)	30.2643(16)	30.2386(18)
b (Å)	8.0668(5)	8.0699(6)
c (Å)	22.2201(12)	22.2267(16)
α (°)	90.00	90.00
β (°)	111.3740(10)	111.369(3)
γ (°)	90.00	90.00
V ($Å^3$)	5051.6(5)	5049.2(6)

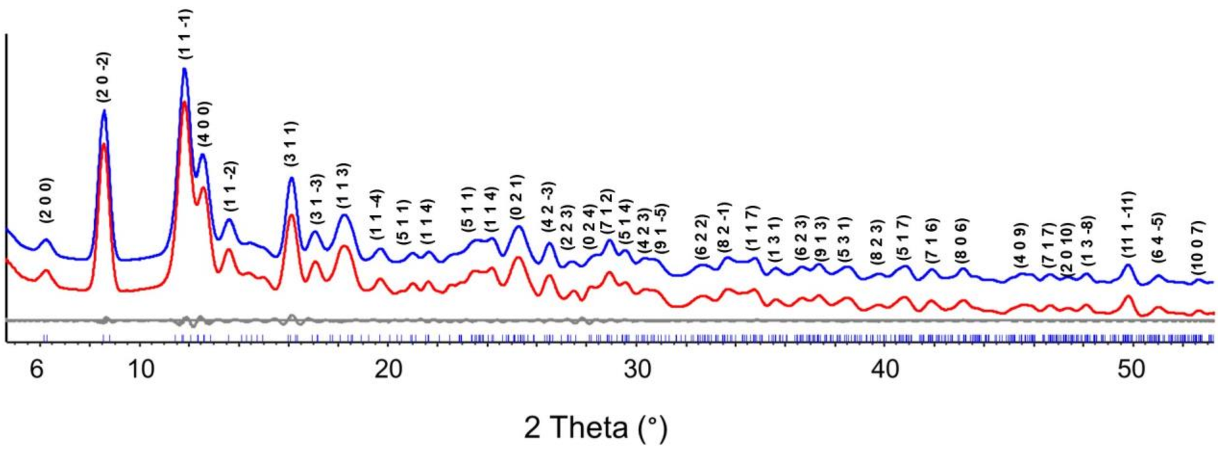

Figure S9. X-ray powder diffraction pattern of **6-Er** and Le Bail fit. The blue and red lines are experimental and calculated patterns, respectively. The grey line is the difference curve with theoretical positions shown at the bottom in blue arrows.

Table S4. Comparison of unit cell parameters for **6-Er** from single crystal data and the Le Bail fit.

	Single crystal data (100 K)	Le Bail fit data (100 K)
Space Group	*C*2	
a (Å)	30.2254(17)	30.2302(18)
b (Å)	8.06230(10)	8.0598(16)
c (Å)	22.2195(6)	22.2210(16)
α (°)	90.00	90.00
β (°)	111.525(5)	111.535(4)
γ (°)	90.00	90.00
V (Å^3)	5037.0(4)	5036.2(6)

V. Crystal Structure Solution and Refinement

Table S5. Crystal data and structure refinement parameters for **1-Y**, **2-La**, **3-Gd**, **4-Tb**, **5-Dy**, and **6-Er**.

Compound	**1-Y**	**2-La**	**3-Gd**
Empirical formula	$C_{64}H_{68}Y_2O_4$	$C_{64}H_{68}La_2O_4$	$C_{64}H_{68}Gd_2O_4$
Formula weight	1079.00	1179.00	1215.68
Temperature (K)	100(2)	100(2)	100(2)
Wavelength (Å)	0.71073	0.71073	0.71073
Crystal system	Monoclinic	Monoclinic	Monoclinic
Space group	*C*2	*C*2	*C*2
a (Å)	30.2969(16)	30.431(3)	30.372(2)
b (Å)	8.0776(4)	8.1136(8)	8.0988(6)
c (Å)	22.2551(11)	22.624(2)	22.3088(16)
α (°)	90.00	90.00	90.00
β (°)	111.4740(10)	109.440(2)	111.0700(10)
γ (°)	90.00	90.00	90.00
V (Å^3)	5068.3(4)	5267.7(9)	5120.5(6)
Z	4	4	4
ρ_{calcd} (g·cm^{-3})	1.414	1.487	1.577
μ (mm^{-1})	2.329	1.648	2.618
F(000)	2248	2392	2448
Crystal size (mm)	0.05×0.19×0.25	0.09×0.13×0.18	0.04×0.06×0.09
θ range for data collection (°)	2.77-29.21	2.84-27.15	2.77-30.11
Reflections collected	72346	51497	64187
Independent reflections	13695 [R_{int} = 0.0438]	11616 [R_{int} = 0.0421]	15039 [R_{int} = 0.0391]
Transmission factors (min/max)	0.6036/0.7458	0.5844/0.7318	0.6872/0.7273
Data/restraints/params.	13695/1/633	11616/235/673	15039/406/709
$R1$,[a] $wR2$[b] ($I > 2\sigma(I)$)	0.0264, 0.0498	0.0410, 0.0950	0.0250, 0.0394
$R1$,[a] $wR2$[b] (all data)	0.0317, 0.0514	0.0461, 0.0977	0.0307, 0.0406
Quality-of-fit[c]	1.041	1.075	1.059

[a] $R1 = \Sigma||F_o|-|F_c||/\Sigma|F_o|$. [b] $wR2 = [\Sigma[w(F_o^2-F_c^2)^2]/\Sigma[w(F_o^2)^2]]$.

[c] Quality-of-fit = $[\Sigma[w(F_o^2-F_c^2)^2]/(N_{obs}-N_{params})]^{½}$, based on all data.

Compound	**4-Tb**	**5-Dy**	**6-Er**
Empirical formula	$C_{64}H_{68}Tb_2O_4$	$C_{64}H_{68}Dy_2O_4$	$C_{64}H_{68}Er_2O_4$
Formula weight	1219.02	1226.18	1235.70
Temperature (K)	100(2)	100(2)	100(2)
Wavelength (Å)	0.71073	0.71073	1.54178
Crystal system	Monoclinic	Monoclinic	Monoclinic
Space group	*C*2	*C*2	*C*2
a (Å)	30.337(3)	30.2643(16)	30.2254(17)
b (Å)	8.0750(7)	8.0668(5)	8.06230(10)
c (Å)	22.305(2)	22.2201(12)	22.2195(6)
α (°)	90.00	90.00	90.00
β (°)	111.2380(10)	111.3740(10)	111.525(5)
γ (°)	90.00	90.00	90.00
V (Å^3)	5093.0(8)	5051.6(5)	5037.0(4)
Z	4	4	4
ρ_{calcd} (g·cm^{-3})	1.590	1.591	1.629
μ (mm^{-1})	2.805	2.946	6.386
F(000)	2456	2464	2480
Crystal size (mm)	0.02×0.11×0.13	0.02×0.06×0.11	0.03×0.05×0.10
θ range for data collection (°)	2.77-31.06	2.81-26.36	3.085-79.396
Reflections collected	67042	57871	45778
Independent reflections	16283 [R_{int} = 0.0462]	10340 [R_{int} = 0.0606]	10202 [R_{int} = 0.0336]
Transmission factors (min/max)	0.5915/0.7462	0.4039/0.4689	0.6042/1.0000
Data/restraints/params.	16283/404/716	10340/649/633	10202/1/633
$R1$,[a] $wR2$[b] ($I > 2\sigma(I)$)	0.0312, 0.0517	0.0324, 0.0427	0.0211, 0.0541
$R1$,[a] $wR2$[b] (all data)	0.0387, 0.0537	0.0454, 0.0477	0.0220, 0.0546
Quality-of-fit[c]	1.033	1.027	1.075

[a] $R1 = \Sigma||F_o|-|F_c||/\Sigma|F_o|$. [b] $wR2 = [\Sigma[w(F_o^2-F_c^2)^2]/\Sigma[w(F_o^2)^2]]$.

[c] Quality-of-fit = $[\Sigma[w(F_o^2-F_c^2)^2]/(N_{obs}-N_{params})]^{½}$, based on all data.

Crystal Growth: All crystals of **1**–**6** were prepared by slow solvent evaporation in the sealed L-shaped glass ampules shown below.

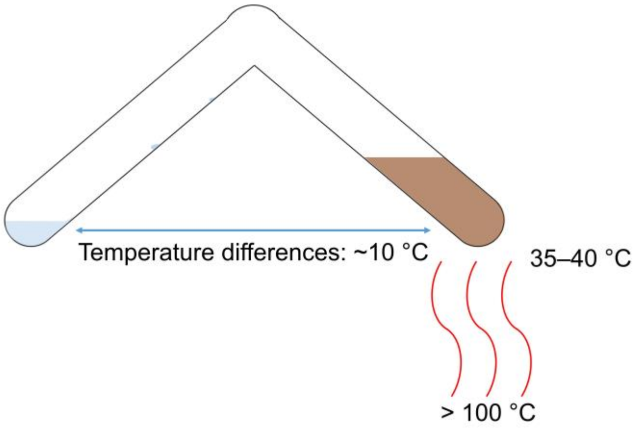

Figure S10. Schematic drawing of sealed L-shaped glass ampules for crystallization of air- and moisture-sensitive compounds.

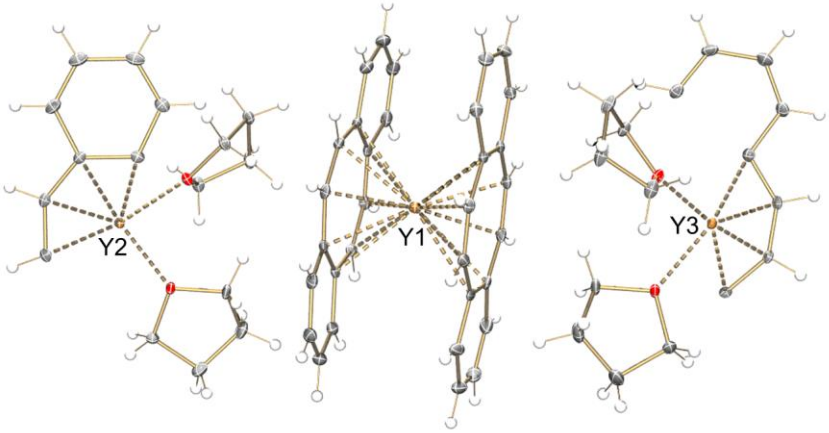

Figure S11. ORTEP drawing of the asymmetric unit of **1-Y** with thermal ellipsoids shown at the 40 % probability level. The color scheme used: C grey, H white, O red, Y copper.

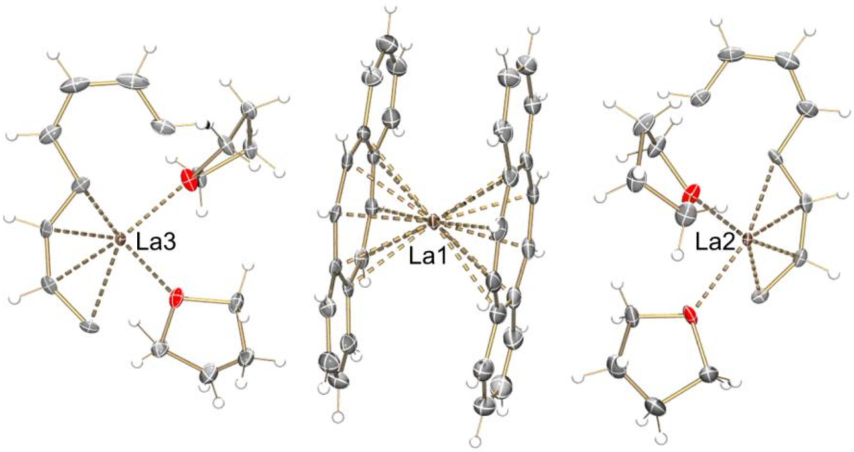

Figure S12. ORTEP drawing of the asymmetric unit of **2-La** with thermal ellipsoids shown at the 40 % probability level. The color scheme used: C grey, H white, O red, La dark-brown.

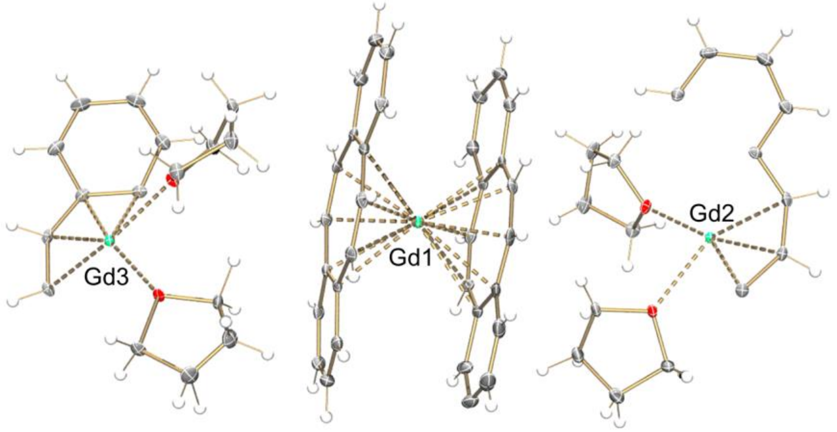

Figure S13. ORTEP drawing of the asymmetric unit of **3-Gd** with thermal ellipsoids shown at the 40 % probability level. The color scheme used: C grey, H white, O red, Gd spring-green.

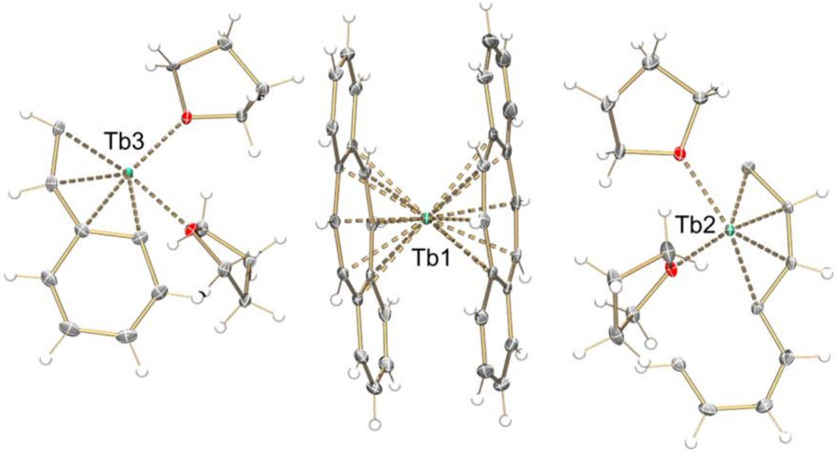

Figure S14. ORTEP drawing of the asymmetric unit of **4-Tb** with thermal ellipsoids shown at the 40 % probability level. The color scheme used: C grey, H white, O red, Tb sea-green.

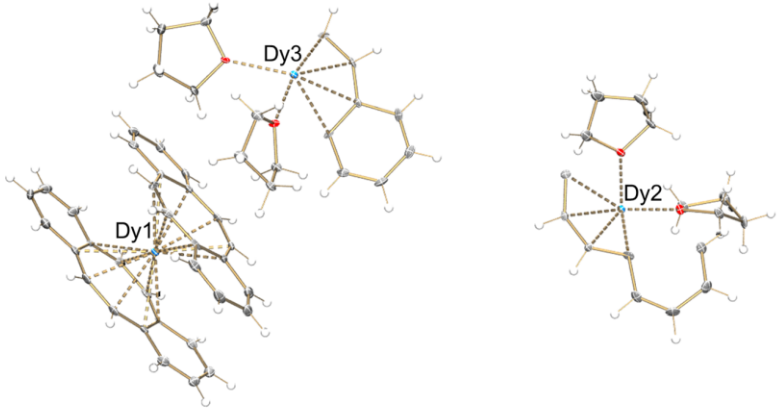

Figure S15. ORTEP drawing of the asymmetric unit of **5-Dy** with thermal ellipsoids shown at the 40 % probability level. The color scheme used: C grey, H white, O red, Dy slate-blue.

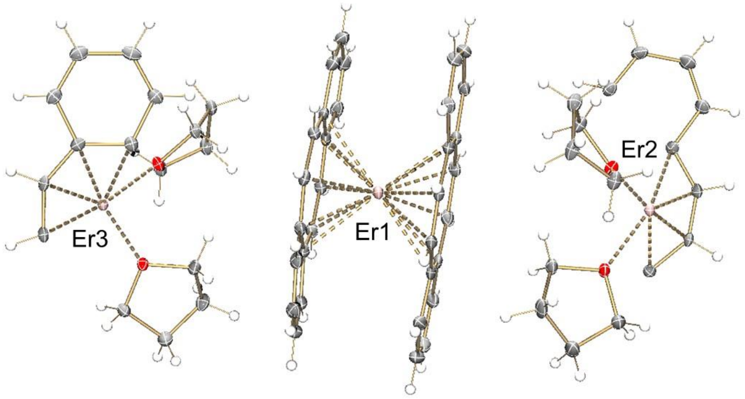

Figure S16. ORTEP drawing of the asymmetric unit of **6-Er** with thermal ellipsoids shown at the 40 % probability level. The color scheme used: C grey, H white, O red, Er pink.

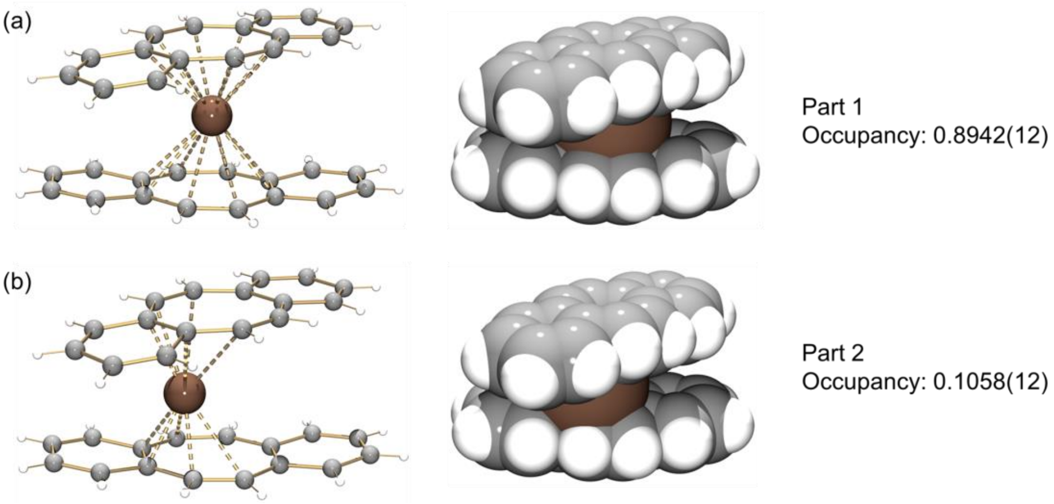

Figure S17. The La-atom disorder in **2-La**, (a) part 1: η^8-coordination, occupancy: 89.4%; (b) part 2: η^5-coordination, occupancy: 10.6%.

VI. Magnetic Properties

Preparation of [RE(DBCOT)(THF)$_4$][RE(DBCOT)$_2$] (RE = 20% Dy and 80% Y) (7-Dy/Y)

A THF (1.0 mL) solution of K_2DBCOT (20 mg, 0.071 mmol) was added to a customized glass system containing a slurry of YI_3 (17.9 mg, 0.038 mmol) and DyI_3 (4.9 mg, 0.009 mmol) in THF (1.0 mL). The reaction mixture was stirred at 25 °C under argon for 24 hours. The initial red color (K_2DBCOT) has changed to brown in 5 hours. The reaction mixture was filtered after 24 hours, and 5.0 mL anhydrous hexanes were added to the filtrate. After 3 hours, the microcrystalline product was collected and packed for magnetic measurements. Yield: 17.3 mg, 60%.

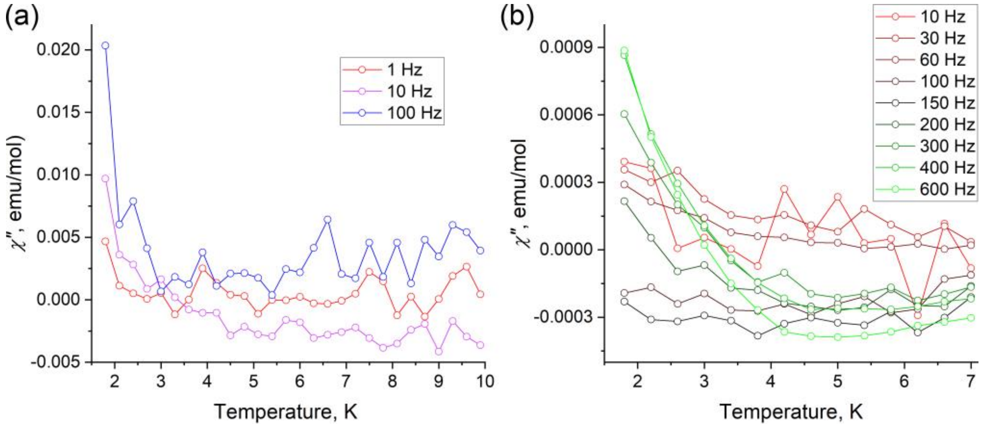

Figure S18. Temperature dependence of out-of-phase AC magnetic susceptibility of **5-Dy** (a) and **7-Dy/Y** (b) measured under zero bias DC magnetic field.

VII. Computational Methods

Throughout the following part, the anionic $[Dy(DBCOT)_2]^-$ system is labeled as **5-a**, and the $[Dy(DBCOT)(THF)_4]^+$ cation is labeled as **5-c**. The positions of hydrogens in **5-a** and **5-c** in **5-Dy** were optimized with Grimme's composite PBEh-3C[1] method using the ORCA electronic structure package, version 5.[2–4] The equilibrium structures were then subjected to single point evaluations at the PBE0[5] and strongly-contracted NEVPT2(9,7)[6–8] levels using the RIJCOSX[9–13] approximation. Scalar relativistic corrections were accounted for with the Zeroth-Order Regular Approximation (ZORA). The lanthanide centers were modeled with the SARC2-ZORA-QZVP[14–17] basis set, and all other atoms were treated with the ZORA-DEF2-TZVP basis set. Natural bond orbital analysis was performed on top of the converged PBE0 single-point evaluations using NBO 7.0.[18]

Magnetic properties were evaluated based on state-averaged CASSCF(9,7) and NEVPT2(9,7) wavefunctions where state-averaged orbitals were used for each state in the NEVPT2 treatments (the so-called canonstep 0 in ORCA parlance). Full intermediate coupling was used for each complex. Spin-orbit coupling was included with quasi-degenerate perturbation theory (QDPT) using the SA-CASSCF wavefunction and strongly contracted NEVPT2 diagonal energies.[19] Ground state multiplet analysis was performed on NEVPT2 results with SINGLE_ANISO[20–22] as implemented in ORCA.

The nephelauxetic reductions (NR) and relativistic nephelauxetic reductions (RNR) for the systems are displayed in Figure S16 and S17. It can clearly be seen that the reductions observed for **5-a** are larger than those observed for **5-c**. This highlights the heightened covalent interactions between the $DBCOT^{2-}$ ligands as compared with the THF ligands. We also note here that this feature is greatly diminished when inspecting the corresponding nephelauxetic reductions based on CASSCF results. It is likely, therefore, that dynamic correlation is an important feature of the $DBCOT^{2-}$/THF-Dy(4f) covalency.

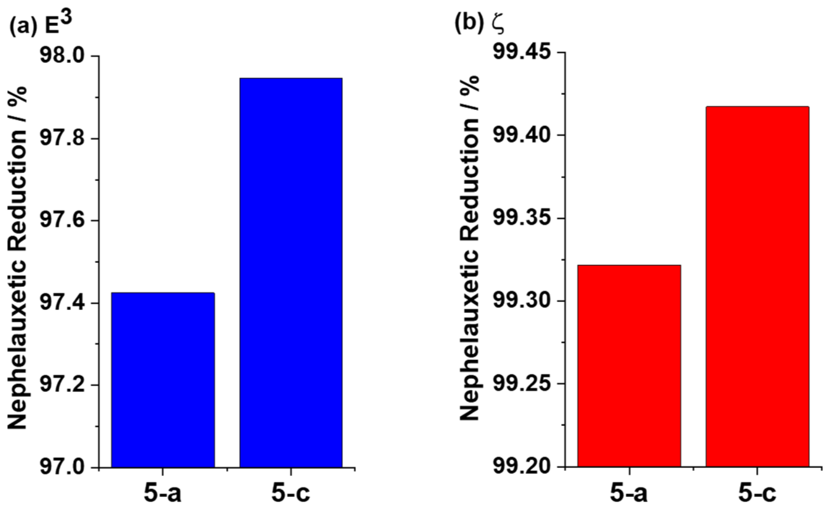

Figure S19. (a) Nephelauxetic reduction and (b) relativistic nephelauxetic reduction at the NEVPT2(*n*,7)/AILFT level.

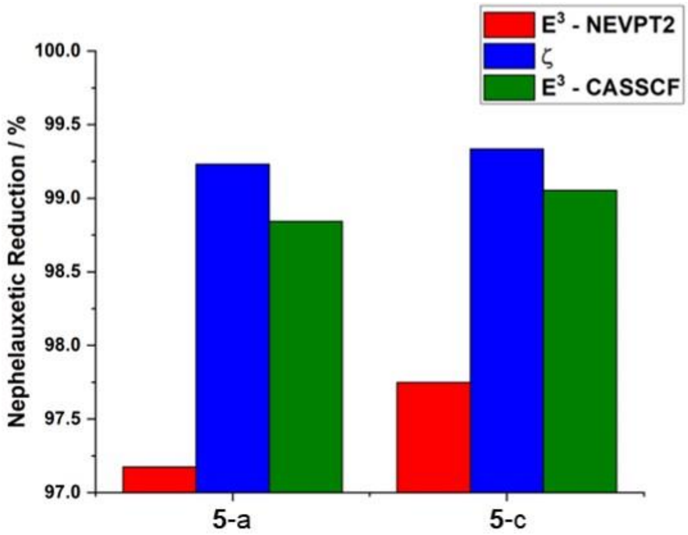

Figure S20. Nephelauxetic reduction (NEVPT2 red, CASSCF green) and relativistic nephelauxetic reduction (blue).

Table S6. Natural population analysis and second-order perturbative stabilizations (kcal/mol) as applied to DFT and SA-CASSCF(9,7) results.

	CASSCF				DFT			
	charge	4f	5d	6s	charge	4f	5d	6s
**5-a**	1.55	9.01	1.25	0.11	1.36	9.12	1.32	0.12
**5-c**	2.03	9.01	0.71	0.12	1.82	9.10	0.79	0.13

Both the DFT and CASSCF results suggest that the strongest interactions are between the DBCOT π-system and the Dy 5d shell. Average Ln-C_{COT} Wiberg bond orders (WBO) of 0.16 are observed for **5-a** at the DFT level, and the corresponding average Dy-C_{COT} WBOs for **5-c** were found to be 0.14. The 5d populations in these systems trends with the donor-acceptor stabilizations between the DBCOT π-system and the 5d shell as determined through second-order perturbation analysis of the Fock matrix in the NBO basis.

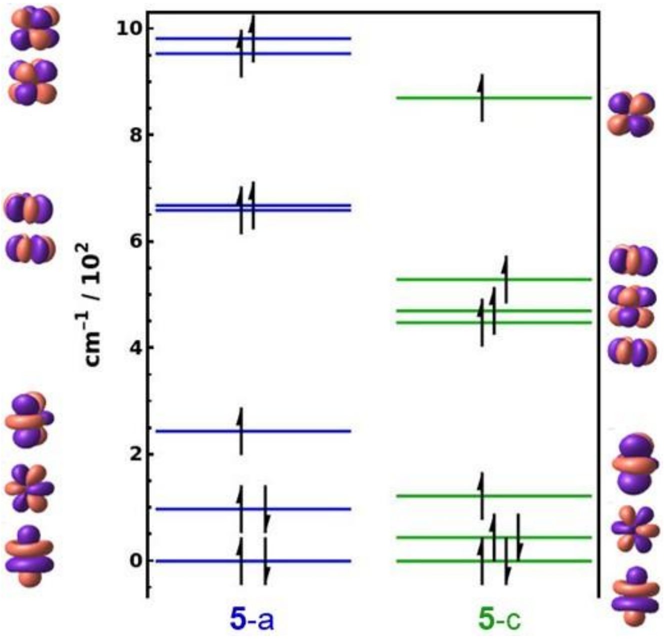

Figure S21. NEVPT2(9,7)/QDPT AILFT f-orbital splitting (blue = **5-a**, green = **5-c**). Orbitals on the left refer correspond to levels in **5-a** and orbitals on right correspond to **5-c**.

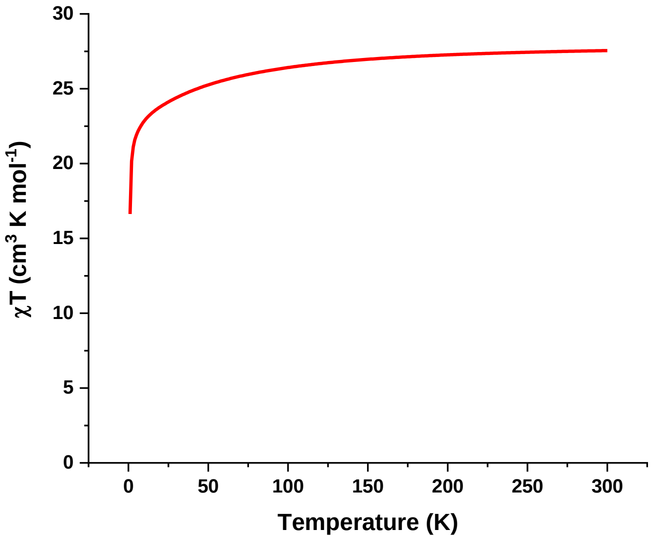

Figure S22. Computed magnetic susceptibility for **5-Dy**.

The computed magnetic susceptibility for **5-Dy** is shown in Fig. S10. Here it can be observed that the room temperature cT product is predicted to be 27.5 cm^3 K mol^{-1}, which is slightly higher than the product observed experimentally. At low temperatures, the computed susceptibility is reduced to 16.6 cm^3 K mol^{-1} at 1K, in similarity to what is observed experimentally.

NEVPT2(9,7)/QDPT/SINGLE_ANISO[20-22] was used to study the ground state multiplets of the systems. The blocking diagrams are shown in Fig. S20. Inspection of Figure S20 shows that there are multiple competing relaxation pathways for **5-a**. We can tentatively attribute this feature to the reduced symmetry present in these systems which strongly mixes different M_J states. This mixing is observed for both **5-a** and **5-c**. Indeed, while the ground state Kramer's doublet (KD) for **5-a** contains > 80% M_J = ±15/2, the second KD contains 10% |±5/2>, 20% |±3/2>, 20% |∓1/2>, and the third KD contains 39% |±13/2>, 16% |±11/2>. For **5-c**, the ground state KD contains > 85% M_J = ±15/2, the second KD contains 16% |±5/2>, 25% |±3/2>, 20% |∓1/2>, and the third KD contains 29% |±13/2>, 15% |±9/2>, 17% |∓1/2>, 11% |∓9/2>.

VIII. References

(1) Grimme, S.; Brandenburg, J. G.; Bannwarth, C.; Hansen, A. Consistent Structures and Interactions by Density Functional Theory with Small Atomic Orbital Basis Sets. *J. Chem. Phys.* **2015**, *143* (5), 054107.
(2) Neese, F. The ORCA Program System. *WIREs Comput. Mol. Sci.* **2012**, *2* (1), 73–78.
(3) Neese, F. *ORCA – An Ab Initio, DFT and Semiempirical SCF-MO Package, Ver. 4.0*; Max Planck Institute for Chemical Energy Conversion: Mülheim a. d. Rurh, Germany, 2017.
(4) Neese, F. Software Update: The ORCA Program System, Version 4.0. *WIREs Comput. Mol. Sci.* **2018**, *8* (1), e1327.
(5) Becke, A. D. A New Mixing of Hartree–Fock and Local Density Functional Theories. *J. Chem. Phys.* **1993**, *98* (2), 1372–1377.
(6) Angeli, C.; Cimiraglia, R.; Evangelisti, S.; Leininger, T.; Malrieu, J.-P. Introduction of N-Electron Valence States for Multireference Perturbation Theory. *J. Chem. Phys.* **2001**, *114* (23), 10252–10264.
(7) Angeli, C.; Cimiraglia, R.; Malrieu, J.-P. N-Electron Valence State Perturbation Theory: A Fast Implementation of the Strongly Contracted Variant. *Chem. Phys. Lett.* **2001**, *350* (3), 297–305.
(8) Angeli, C.; Bories, B.; Cavallini, A.; Cimiraglia, R. Third-Order Multireference Perturbation Theory: The n-Electron Valence State Perturbation-Theory Approach. *J. Chem. Phys.* **2006**, *124* (5), 054108.
(9) Neese, F. An Improvement of the Resolution of the Identity Approximation for the Formation of the Coulomb Matrix. *J. Comput. Chem.* **2003**, *24* (14), 1740–1747.
(10) Neese, F.; Wennmohs, F.; Hansen, A.; Becker, U. Efficient, Approximate and Parallel Hartree–Fock and Hybrid DFT Calculations. A 'Chain-of-Spheres' Algorithm for the Hartree–Fock Exchange. *Chem. Phys.* **2009**, *356* (1), 98–109.
(11) Kossmann, S.; Neese, F. Comparison of Two Efficient Approximate Hartee–Fock Approaches. *Chem. Phys. Lett.* **2009**, *481* (4), 240–243.
(12) Izsák, R.; Neese, F. An Overlap Fitted Chain of Spheres Exchange Method. *J. Chem. Phys.* **2011**, *135* (14), 144105.
(13) Ganyushin, D.; Gilka, N.; Taylor, P. R.; Marian, C. M.; Neese, F. The Resolution of the Identity Approximation for Calculations of Spin-Spin Contribution to Zero-Field Splitting Parameters. *J. Chem. Phys.* **2010**, *132* (14), 144111.
(14) Aravena, D.; Neese, F.; Pantazis, D. A. Improved Segmented All-Electron Relativistically Contracted Basis Sets for the Lanthanides. *J. Chem. Theory Comput.* **2016**, *12* (3), 1148–1156.
(15) Chmela, J.; Harding, M. E. Optimized Auxiliary Basis Sets for Density Fitted Post-Hartree–Fock Calculations of Lanthanide Containing Molecules. *Mol. Phys.* **2018**, *116* (12), 1523–1538.
(16) Weigend, F. Accurate Coulomb-Fitting Basis Sets for H to Rn. *Phys. Chem. Chem. Phys.* **2006**, *8* (9), 1057–1065.
(17) Weigend, F.; Ahlrichs, R. Balanced Basis Sets of Split Valence, Triple Zeta Valence and Quadruple Zeta Valence Quality for H to Rn: Design and Assessment of Accuracy. *Phys. Chem. Chem. Phys.* **2005**, *7* (18), 3297–3305.
(18) Glendening, E. D.; Badenhoop, J. K.; Reed, A. E.; Carpenter, J. E.; Bohmann, J. A.; Morales, C. M.; Karafiloglou, P.; Landis, C. R.; Weinhold, F. *NBO 7.0*; Theoretical Chemistry Institute, University of Wisconsin: Madison, WI, 2018.

(19) Ganyushin, D.; Neese, F. First-Principles Calculations of Zero-Field Splitting Parameters. *J. Chem. Phys.* **2006**, *125* (2), 024103.
(20) Chibotaru, L. F.; Ungur, L. Ab Initio Calculation of Anisotropic Magnetic Properties of Complexes. I. Unique Definition of Pseudospin Hamiltonians and Their Derivation. *J. Chem. Phys.* **2012**, *137* (6), 064112.
(21) Iwahara, N.; Ungur, L.; Chibotaru, L. F. ˜J-Pseudospin States and the Crystal Field of Cubic Systems. *Phys. Rev. B* **2018**, *98* (5), 054436.
(22) Ungur, L.; Chibotaru, L. F. Ab Initio Crystal Field for Lanthanides. *Chem. – Eur. J.* **2017**, *23* (15), 3708–3718.
